# Ileum tissue single-cell mRNA sequencing elucidates the cellular architecture of pathophysiological changes associated with weaning in piglets

**DOI:** 10.1186/s12915-022-01321-3

**Published:** 2022-05-30

**Authors:** Wenjie Tang, Yifan Zhong, Yusen Wei, Zhaoxi Deng, Jiangdi Mao, Jingliang Liu, Teresa G. Valencak, Jianxin Liu, Heping Xu, Haifeng Wang

**Affiliations:** 1 College of Animal Science, Zhejiang University, The Key Laboratory of Molecular Animal Nutrition, Ministry of Education, Hangzhou, 310000 China; 2 Key Laboratory of Growth Regulation and Translational Research of Zhejiang Province, School of Life Sciences, Westlake University, Hangzhou, 310000 Zhejiang China

**Keywords:** Single-cell mRNA-sequencing, Ileum, Weaned reaction, Piglet

## Abstract

**Background:**

In mammals, transitioning from sole milk uptake to the intake of solid feed results in dramatic developmental changes in intestinal function and immunological status. In fact, weaning stress is often accompanied by intestinal inflammatory processes. To develop effective intervention strategies, it is necessary to characterize the developmental pattern and immune response that occurs on weaning, as we have done in this study for piglets.

**Results:**

To comprehensively delineate cell heterogeneity in ileum tissues and the underlying mechanisms in weaning-induced intestinal inflammation of piglets, we have analyzed the transcriptomes of 42,149 cells from ileum mucosa of normally suckling and post-weaned piglets. There were 31 cell subtypes including epithelial, stromal, and immune cells. A bifurcating trajectory was inferred to separate secretory and absorptive lineages. Integrated cross-species datasets showed well-conserved cellular architectures and transcription signatures between human and pig. Comparative analyses of cellular components, cell–cell communications, and molecular states revealed that T cell subpopulations were significantly altered in weaned piglets. We found that T helper (Th) 17 functional plasticity across changes in the cytokine milieu and the enrichment of granzyme B (*GZMB*)-expressing cytotoxic T cells potentially exacerbate mucosal inflammation via mitochondrial dysfunction in epithelial cells.

**Conclusions:**

Our work has elucidated the single-cell molecular characteristics of the piglet ileum before and after weaning. We have provided an atlas that portrays the landscape of the intestinal pathophysiological inflammatory process associated with weaning, finding a level of conservation between human and pig that support the use of piglets as a model for human infants.

**Supplementary Information:**

The online version contains supplementary material available at 10.1186/s12915-022-01321-3.

## Background

The termination of lactation with simultaneous introduction and adaptation of semisolid or solid foods in suckling neonates is ultimately necessary for meeting nutritional and metabolic requirements later in life. The weaning process in the young remains extraordinary challenging and requires maintenance of the equilibrium between maternal investment into lactation and the nutritional effects on the offspring [1]. Studies from humans indicate that delayed weaning can prolong the infancy-childhood transition period, which may have lifelong effects on incidence of overweight individuals [2]. Similarly, the weaning process affects intestinal development and maturation in rats [3] and is exposing to multiple even more detrimental conditions, including the loss of mother-derived immunological support, insufficient maturity of the digestive system, and low voluntary food intake [1, 4, 5]. These changes are frequently associated with increased risks for serious intestinal morphological damage and enteric inflammation [3, 6].

The weaning strategy and its consequences have been extensively investigated in piglets [7, 8]. Weaned piglets exhibit similar gastrointestinal physiological responses as those observed in human infants, including transient anorexia, diarrhea, and inefficient nutrient utilization, during the transition from normal suckling to solid feed intake. Therefore, weaned piglets may serve as a meaningful model for studying weaning stresses in human infants [9]. Although the effects of weaning on the physiological and local immune system within the gastrointestinal tract have been studied before, most published studies have focused on whole tissues or bulk cell populations [10, 11]. To our knowledge, no comprehensive single-cell landscape has been profiled in piglet intestines yet. Similarly, exact details of the intestinal architecture and cell type-specific molecular changes during weaning are lacking. In particular, the extensive degree of intestinal cellular heterogeneity requires accurate identification of molecular and functional properties of cell subtypes from bulk-mRNA sequencing [12, 13].

Single-cell RNA-sequencing (scRNA-seq) enables systematic elucidation of cellular components, cell lineage trajectories, and their gene signatures. Despite emerging information on cell heterogeneity from mouse and human gastrointestinal tissues and immune niches during physiological and pathological conditions [14–16], to our knowledge, no detailed studies have been describing transcriptional profiles of intestinal tissues at single-cell level in weaned mammals. Piglets are anatomically, physiologically, and genetically similar to humans and represent an excellent non-rodent model for human infants [17, 18]. Single-cell transcriptome profiling facilitates a systematic, comparative analysis of cell types between humans and pigs, which provides valuable insights into the utilization of porcine models for future translational studies. Deciphering intestinal development and immune reactions during stress induced by weaning may contribute to develop powerful intervention strategies.

The small intestinal epithelium has an unusually high turnover rate (4- to 5-day time scale) [19]. This time window however enables to observe the entire process of epithelial compartment renewal during the transition from milk suckling to the uptake of solid food in piglets. Here, we present a scRNA-seq map of 42,149 cells from the ileum tissues from normally suckling piglets (NSPs) and post-weaned piglets (PWPs). We comprehensively delineated various differentiated cellular subtypes within the ileum mucosa, mainly including absorptive lineage cells, secretory lineage cells and immune cells. Cross-species pairwise comparative analyses were performed to investigate common and divergent features of ileal epithelial cells and immune cells from humans, mice and pigs. Comparative analyses showed the reduced levels of T helper (Th)17 cells and increased fractions of cytotoxic T lymphocytes (CTLs) in PWP, compared with those in NSP. Th17 cells in PWPs were overexpressing *TNF-α* and primarily represented pathogenic phenotypes. The transcriptomic status of Th17 may be affected by the special cytokine environment according to recent studies [20, 21]. Our results also suggest that enrichment of *CD8*^+^ *GZMB*^+^ CTLs in PWPs may induce epithelial cell apoptosis and ultimately may lead to severe intestinal inflammation. Thus, our work sheds light on the unique single-cell molecular characteristics of the piglet ileum, strengthening our understanding of the intestinal pathophysiological inflammatory process during weaning. This study offers insights into developing new therapeutic strategies to mitigate weaning disorders. We present a suitable pig model for studying stress-induced gastrointestinal imbalances during childhood.

## Results

### Comprehensive single-cell transcriptional atlas of the piglet ileum

**Fig. 1:**
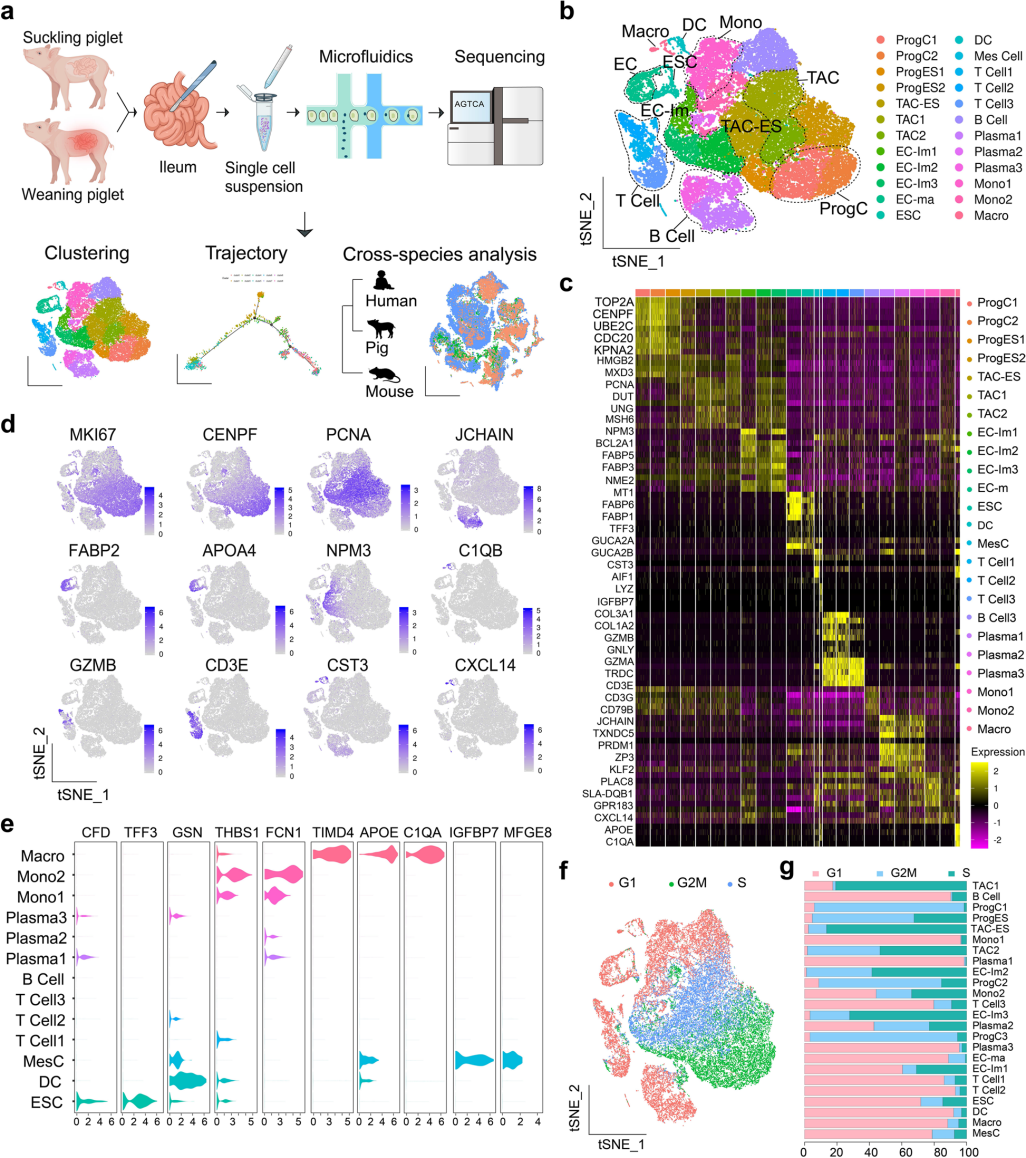
Single-cell transcriptome analyses uncover cell composition of ileum tissues in piglets. **a** Schematic of the sample collection, scRNA-seq, and cross-species analytic workflow. **b** t-Stochastic neighbor embedding (t-SNE) plots of all cell clusters obtained from NSPs and PWPs (*n* = 3), showing 24 distinct cell types identified from the first clustering. EC, enterocyte (Im: immature, Ma: mature); ESC, epithelial secretory cell; ProgC, progenitor cell; TAC, transient-amplifying cell; Macro, macrophage; DC, dendritic cell; Mono, monocyte; Mes, mesenchyme. **c** Heatmap showing the differential expression of cell type-specific genes in intestinal structural and immune cells. **d** t-SNE plots showing expression levels of canonical markers for various intestinal epithelial and immune cell populations. The expression intensity is indicated by the purple color. The expression scale is normalized by the logNormalize method in Seurat. **e** Violin plots depicting expression levels of representative marker genes across Macro, DC, Mono, and ESC clusters. The *y*-axis shows the normalized gene expression values. See Additional file 1: Table S1 for all marker genes. **f** t-SNE plots with single cells colored according to their cell-cycle phase. **g** Histogram showing cell-cycle quantity of epithelial, stromal, and immune cell subsets in piglet ileum mucosa

We aimed to explore cellular heterogeneity and characteristic changes associated with weaning in ileum of PWP. We dissected ileum tissues for single cell isolation from each experimental group following a standard protocol and performed scRNA-seq using a 10× Chromium Genomics pipeline (Fig. 1a). We obtained approximately 6500–8600 cells per sample after computational quality control and surrounding RNA removal. A total of 42,149 high-quality cell profiles yielded 24 preliminary clusters using *t*-distributed stochastic neighbor embedding (t-SNE) (Fig. 1b) [22].

Canonical marker genes were used to identify several distinct clusters (Fig. 1c; Additional file 1: Table S1), including enterocytes (ECs), epithelial secretory cells (ESCs), mesenchymal cells, and immune cells (Fig. 1d, e). Ileum epithelial cells (60.54%) and immune cells (27.15%) were the most abundant cell types in our dataset, which is in accordance with previously described cellular compositions from small intestine tissues [23]. At least four different states were identified in the intestinal epithelial cell lineage (Fig. 1b). Common mature EC markers included genes relating to nutritional digestion and absorption, such as *FABP6*, *MT1A*, *FABP1*, *FABP2*, *APOA1*, and *APOA4* [12, 13], and the glutathione metabolism signaling gene *ANPEP* [24]. Immature ECs were characterized by the concerted expression of marker genes *FABP3*, *PCNA*, *MCM4*, and *NPM*, indicating distinct stages of maturation [14, 25]. Well-known cell-type markers for progenitor cell (ProgCs) such as *MKI67*, *TOP2A*, *CENPF*, and *UBE2C* [26] and *PCNA* and *MCM6* for transient-amplifying cells (TACs) [27] were used to identify respective clusters. We differentiated 5 major immune cell types, among which B and T lymphocytes expectedly remained the most abundant components. The B cell clusters were characterized by the expression of *MS4A1*, *CD79A*, *CD79B*, *MZB1*, and *JCHAIN* [28, 29]. T lymphocytes highly expressed the cell-surface molecular gene *CD3D*/*E*/*G* and co-expressed unique gene sets, such as *PDCD1*, *GLNY*, and *GZMB*, possibly reflecting differential, activated, or resting states, respectively [30]. In our dataset, 3 other immune cell clusters were identified by differential expression of canonical markers: monocytes (*FCN1*, *THBS1*, and *APOBEC3B*) [29], macrophages (*C1QA*/*B*/*C*), and dendritic cells (DCs; *CCR7*, *CST3*, and *CD103*) [31] (Fig. 1e, Additional file 1: Table S1). The central Cluster 10 (Additional file 2: Fig. S1a) failed to express any distinct marker genes. Next, we further investigate cluster-specific gene features using Gene Ontology (GO) analysis. Cluster 10 showed pronounced expression of genes relating to polarized epithelial morphogenesis and genes associated with the regulation of monocyte differentiation; therefore, we assigned Cluster 10 to monocytes (Additional file 1: Table S1). Cluster 20 comprised a variety of cells with non-overlapping marker gene expression for diverse ESC types, including markers for enteroendocrine (*CHGA*) and goblet (*TFF3*) cells and novel cell markers (*BEST4* and *OTOP2*). Therefore, we classified Cluster 20 as ESCs. A group of comparatively rare mesenchymal cells were identified in the piglet ileum samples, characterized by exclusive expression of *COL3A1*, *COL1A2*, and *COL1A1* [32]. Overall, annotated subsets were reproducible, with almost all cell types identified in all samples and proportional distributions across individual piglets (Additional file 2: Fig. S1a, b). Based on primary clustering, we observed no significant difference in the percentages of major intestinal epithelial or innate immune cells between NSPs and PWPs (Additional file 2: Fig. S1c). We also performed a cell-cycle analysis and visualized every single cell from transitioning cell-cycle states (Fig. 1f). Gene sets from each cell-cycle phase were differentially expressed in all cell subtypes (Fig. 1g). We used the FindVariableFeatures function to confirm that cell-cycle-related genes were not the core drivers of variation in the PCA analysis (Additional file 2: Fig. S1d, e). ProgCs and TACs were characterized by high expression rates of gene sets relating to G2M and S phase and by low expression in G1 phase genes (Fig. 1g). Both ProgCs and TACs are key proliferative cells and responsible for replenishing intestinal epithelium.

### The heterogeneity and similarity of ESC subgroups

**Fig. 2:**
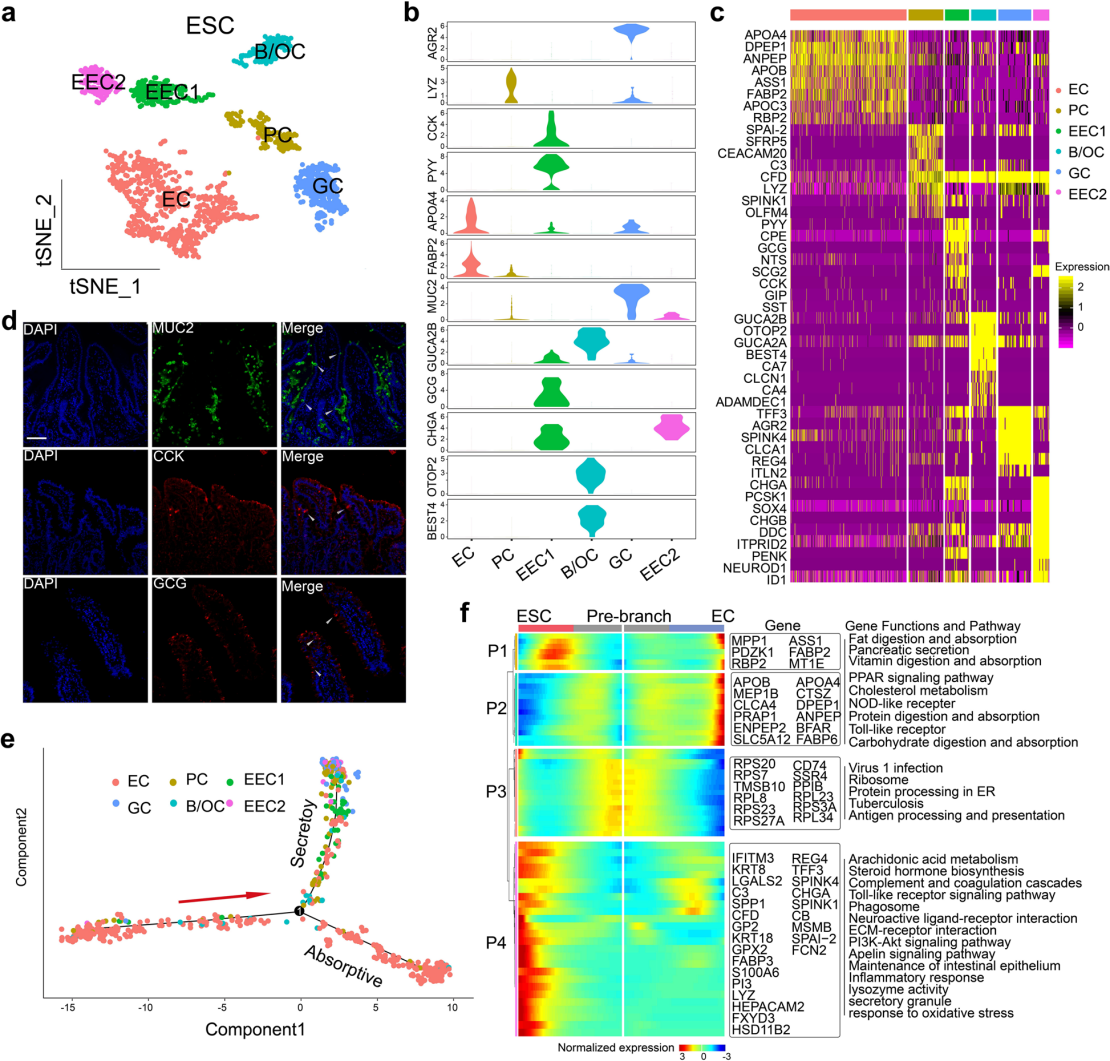
Heterogeneity of epithelial secretory cell subtypes in ileum tissues of piglets. **a** t-SNE plot displaying 832 epithelial secretory cells (ESCs) separated into 6 subtypes. EC, enterocyte; GC, goblet cell; EEC, enteroendocrine cell; B/OC, *BEST4*/*OTOP2* cell. **b** Violin plots showing canonical marker genes across ESC subtypes. See Additional file 3: Table S2 for all marker genes. **c** Heatmap reflecting differentially expressed transcription factors and cell-type-specific genes in ESC subtypes. **d** Immunofluorescence (IF) staining assay of GC marker *MUC2* (green) and EEC markers *CCK* (red) and *GCG* (red) (*n*=6); arrow, target cell type; scale bars, 50 μm. **e** Differentiation pseudotime trajectory analysis of ESC subtypes. Predicted secretory lineage cells include GC, EEC, and B/OC; enterocytes (EC) represent absorptive cells. Red arrow indicates the direction of differentiation. **f** The differentially expressed genes (rows) along the pseudotime (columns) of secretory and absorptive cells clustering hierarchically into four profiles. The representative gene functions and pathways of each profile are shown

The primary analysis of cellular subpopulations and gene profiles allowed us to classify and define putative ESC subtypes. Clustering analysis divided ESCs into six subsets with distinct expression signatures including Paneth cells (PC), goblet cells (GC), enteroendocrine cells (EEC), *BEST4*/*OTOP2* cells (B/OC), and ECs (Fig. 2a). A group of PCs was identified with specific expression of *LYZ* in the ileum (Fig. 2b, c and Additional file 3: Table S2). We discovered a cluster of 134 cells as GCs according to the canonical marker genes *MUC2* (Fig. 2b), *TFF3*, and *AGR2*C (Fig. 2c, Additional file 3: Table S2) [23]. *CHGA*, as the marker of EEC, was highly expressed in the clusters of EEC1 and EEC2. *NEUROD1* and *SOX4*, considered as typical EEC precursor markers [13], were highly expressed in EEC2 (Fig. 2c, Additional file 3: Table S2 and Additional file 4: Fig. S2a). EEC1 highly expressed mature EEC marker genes such as secretory granules, peptide hormone processing genes, and hormone activity related genes (Additional file 4: Fig. S2b and Additional file 5: Table S3). The presence and anatomical location of *MUC2*^+^ GCs, *CCK*^+^ EECs, and *GCG*^+^ EECs were confirmed in ileum samples via immunofluorescence (IF) staining assays (Fig. 2d).

Our results revealed a sub-cluster of ESCs that distinctly expressed *BEST4* and *OTOP2/3* [33], which are involved in electrolyte balance and underlie perception of sour taste and pH sensing [34]; these cells were designated as B/OCs (Fig. 2a, b). Another signature of the B/OC cluster was enriched in the satiety peptide uroguanylin and an endogenous paracrine hormone (encoded by *GUCA2B*; Fig. 2c), which participates in the positive regulation of guanylate cyclase activator activity [35]. Functionally, GO enrichment analysis of differentially expressed genes (DEGs) highlighted potential processes involved in the release of sequestered calcium ions into the cytosol, lipoprotein transporter activity, and the regulation of exocytosis (Additional file 4: Fig. S2c). After identifying ESC subgroups, we found that all of these cell subtypes were shared between NSPs and PWPs, with no substantial differences (Additional file 4: Figs. S2d, e).

To distinguish cell lineages during the differentiation of several ESC subsets, unsupervised pseudotime analysis was performed to identify trajectory bifurcation points (Fig. 2e). ECs were found in the beginning of the trajectory path, whereas the secretory lineage, including PC, GC, EEC, and B/OC, was observed in a terminal state [34]. Unexpectedly, PC, GC, EEC, and B/OC were intermixed on the trajectory of the secretory lineage due to the general similarity among their transcriptomes. These results showed the degree of heterogeneity among secretory cell in the piglet ileum and the existence of parallel differentiation hierarchies across PCs, GCs, EECs, and B/OCs. We further determined the different expression patterns of genes along the trajectory of ESC subgroup differentiation. We identified several critical genes that were specifically expressed at continuous pseudotimes on the trajectory map, such as *PYY* in EEC1, *CHGA* in EEC2, *FCN2* and *SPINK4* in GC, and *GUCA2A* in B/OC, respectively (Additional file 4: Fig. S2f). These transcription factors may play important roles for cell fate in the corresponding subgroups. Additionally, the top 50 genes with significant dynamic expression changes were displayed from the anterior to posterior segments of each branch (Fig. 2f). A branched heat map revealed four gene expression patterns (P1–P4) with dissimilar transcriptional changes associated with transitional states. The functional enrichment analysis displayed that genes relating to digestion and absorption, to pancreatic secretions, and to cholesterol metabolism gradually had increased expression levels in ECs and reduced expression levels in ESCs when entering the terminal states (P1 and P2, Fig. 2f, Additional file 6: Table S4). In the P3 phase, we detected a dynamic trend in gene expression with the highest level at the pre-branch stage. In the ESC branch, these genes were gradually downregulated before they were increased during the terminal state. They were consistently downregulated across the pseudotime map in the EC branch. The 12 genes in P3 encode ribosomal protein family members, which encode structural constituents of ribosomes and contribute to biosynthetic functions [36]. In the P4 phase, multiple classical secretory signaling pathways were activated during ESC differentiation, including steroid hormone biosynthesis, phosphoinositide 3-kinase (PI3K)-protein kinase B (AKT), and secretory granules, and activation increased towards the terminal stage. By contrast, genes associated with these functions were lost when transiting into the absorptive state. These results suggest different developmental events in secretory and absorptive cells, in which differentiation might be controlled by the suppression of ribosomal genes and the activation of specific lineage factors.

### Distinct subgroups, transition trajectories, and resident states of immune cells in NSPs and PWPs

**Fig. 3:**
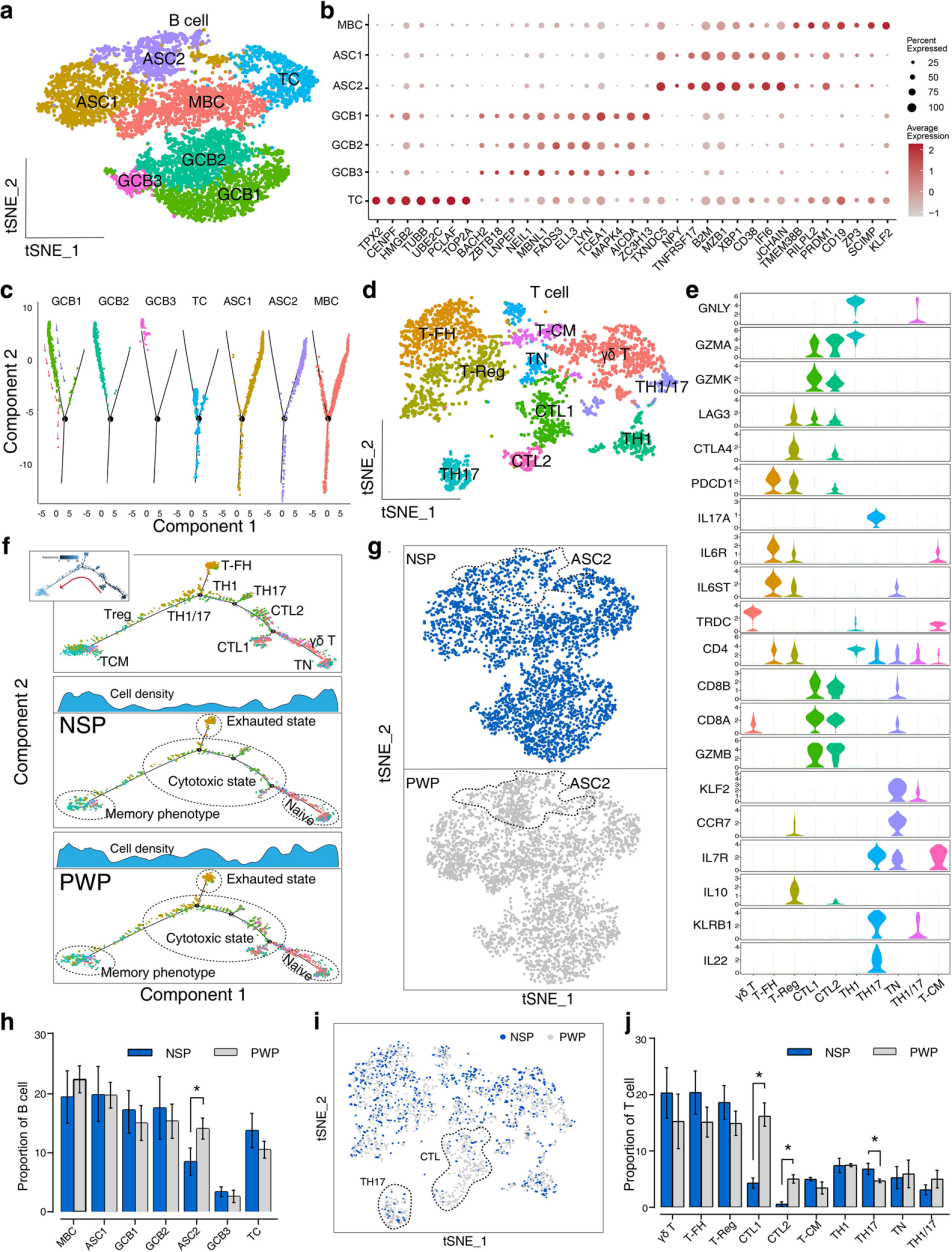
Heterogeneity of immune cell subtypes in ileum tissues of piglets. **a** t-SNE plot showing representations of B cell subclusters. MBC, memory B cell; ASC, antigen-secreting cells; GCB, germinal-center B cell; TC, transitory cell. **b** t-SNE plot showing representations of T cell subclusters. Th, T helper; Tn, T naïve; Tfh, T follicular helper; TCM, T central memory; Treg, T regulatory; CTL cytotoxic T lymphocytes. **c** Violin plots showing canonical marker genes across T cell subtypes. **d** Dot plot showing the scaled expression of signature genes for each B cell subtype, colored by the average gene expression. Dot sizes reflect the percentage of cells in each cluster expressing the corresponding gene. See Additional file 7: Table S5 for all marker genes. **e** Pseudotime-ordered analysis of T cell subtypes from NSP (middle panel) and PWP (down panel). T cell subpopulations are marked by the colors in the top panel. The distribution of cell density with various state is shown at the top. **f** Pseudotime-ordered analysis of B cell subtypes. Each subpanel corresponds to a previously identified cell type (**a**). **g** t-SNE plot showing cell counts for B cell subtypes from NSPs and PWPs. Dashed line, differences in ASC2 cell numbers between NSPs and PWPs. **h** Changes in cell proportions of B cell subtypes. Error bars: SEM; *FDR < 0.025, Wald test. **i** t-SNE plot showing T cell subtype counts from NSPs and PWPs. Dashed line, differences in Th17 and CTL cell numbers between NSPs and PWPs. **j** Changes in cell proportions for T cell subtypes. Error bars: SEM; *FDR<10^−4^, Wald test

To better explore the immune niche cells, we performed re-clustering analysis of T cells and B cells individually. Seven clusters identified from B cells were designated as memory B cells (MBC), antigen secretory cells (ASC), germinal-center B cells (GCB), and transitory cells (TC) (Fig. 3a, b, Additional file 7: Table S5). Specifically, the MBC cluster was characterized by high expression levels of *SCIMP*, *CD19*, and *KLF2* [37, 38]. The two ASC clusters highly expressed *CD38*, *MZB1*, *XBP1*, and *SDC1* [39], whereas the three GCB clusters highly expressed *ZC3H13*, *AICDA*, and *TCEA1* (Fig. 3b, Additional file 8: Fig. S3a). The TC cluster displayed a hybrid transcriptomic signature with other B cell subtypes and expressed multiple proliferation genes (*TOP2A*, *TUBB*, and *HMGB2*; Fig. 3b, Additional file 8: Fig. S3b).

To analyze the dynamic transition of B cells, we performed a trajectory analysis for cell subsets (Fig. 3c). The results were then separated into seven subpanels embedded with gradually transiting subsets from GCB to ASC, with additional TCs gathering at the central branch point, and MBCs being spread over the ASC lineage (Fig. 3c). We found primary transcriptional changes associated with transitions, and *BCL6*, *ELL3*, and *PRRT1B* (promoting confinement to germinal centers) [40] were highly expressed in GCBs and silenced in ASCs. Conversely, genes related to the endoplasmic reticulum (ER) membrane and ubiquitin-dependent ER-associated degradation pathway (associated with antigen processing and presentation) [41] were activated during ASC progression (Additional file 8: Fig. S3c and Additional file 9: Table S6).

We performed additional sub-clustering analyses in 3242 T cells to disentangle the high diversity of T cell subsets. T cells could be separated into 10 subpopulations (Fig. 3d). Signature genes, such as *TRDC*, *GNLY*, *IL17A*, *KLF2*, *IL6ST*, *IL10*, and *IL7R*, were among the most significant DEGs identified among γδ T, Th1, Th17, T naïve (Tn), T follicular helper (Tfh), T regulatory (Treg) cells, and T central memory (TCM), respectively [30, 42] (Fig. 3e, Additional file 8: Fig. S3e and Additional file 10: Table S7). In the ileum mucosa, two clusters simultaneously expressed cytotoxic and effector markers (*CCL3L1*, *CD8A*, *GZMB*, and *GZMK*) and moderately expressed exhaustion-associated molecules (*CTLA4* and *LAG3*), implying that these cells were CTLs [43–45] (Additional file 11: Fig. S4a, b). The dynamic immune states of T cells were subjected to pseudotime analysis to identify the trajectories of Tn cells into memory phenotypes through an intermediate cytotoxic or exhausted state (Fig. 3f). We compared the cell density distribution, which was aligned across pseudotime in both NSPs and PWPs. The results showed that cytotoxic and exhausted cell states were predominantly distributed in PWPs relative to NSPs, whereas no other T cell types displayed visible differences between the two groups. We further identified genes as being significantly differentially expressed among the subpopulations in Tn and CTL (Additional file 11: Fig. S4c). In addition to different signatures associated with cytotoxic capacities (granzymes, perforin, and chemokine ligands), we observed other potential signals for activating T cells, such as a high expression of killer cell lectin-like receptor (*KLR*) and *CRTAM*. Genes from the cell-cycle and high mobility group family (*HMGF*) were consistently silenced.

To delineate proportional changes in the cellular compositions of B and T cell subtypes between NSPs and PWPs, we compared their scRNA-seq profiles using t-SNE analysis and confirmed significant differences, in accordance with a previous method [13]. The general distribution patterns of B subtypes were similar between NSPs and PWPs, whereas the proportion of ASC2 was significantly increased in PWPs (Fig. 3g, h). The ASC2 cluster was characterized by a significantly enriched signaling pathway participating in the intestinal immune network for IgA production and antigen processing and presentation (Additional file 8: Fig. S3d). We performed multiplexed IF staining for *CD45*, *SDC1*, and *IGA* to validate scRNA-seq data. *SDC1*^+^ *IGA*^+^ ASCs were found to reside in intestinal villi and to be highly abundant in the PWP samples (Additional file 12: Fig. S5). These results indicate that ASC2 cells might implement mucosal antibody responses, mediating intestinal inflammation [46]. We observed that the composition of T cell subpopulations differed remarkably between NSPs and PWPs (Fig. 3i, j), in line with the observed cell densities in the transition trajectory. Specifically, the percentages of CTLs were significantly increased in PWPs compared with NSPs, whereas Th17 cells were reduced. These results suggested that the unique immune ecosystem (especially for T cells) in PWPs may be associated with inflammatory features caused by stress due to weaning [7].

Besides examining adaptive immunity, we also performed a comparative analysis for innate-like immunity, such as macrophages and monocytes. Within a single macrophage cluster, we detected 176 differentially expressed genes from the NSP and PWP group (Additional file 13: Fig. S6a). Through a GO analysis of these differentially expressed genes (Additional file 13: Fig. S6b), we found higher levels of T cell mediated cytotoxicity, macrophage colony-stimulating factor, and antigen process and presentation pathway in macrophages. However, no comparable difference was observed in monocytes from the two groups, when comparing the expression pattern of 14,423 genes (Additional file 13: Fig. S6c).

### Across-species comparison analysis of cell-types in the ileum epithelial and immune cell atlas

**Fig. 4:**
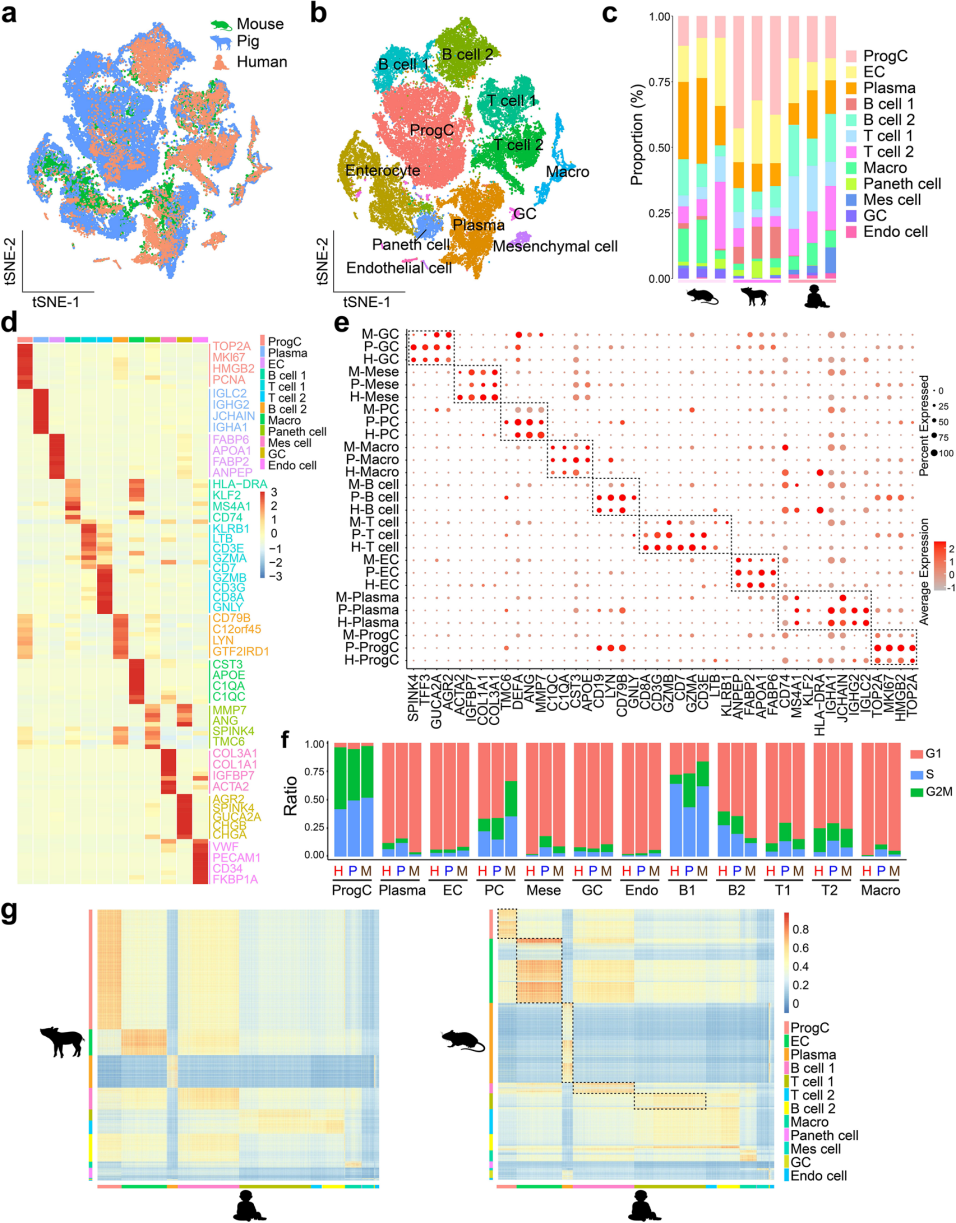
Across-species comparison of cellular diversity and gene expression profiles of ileum. **a** t-SNE plots showing the datasets alignment of ileum cells from the humans (*n* = 11,042), pigs (*n* = 18,754), and mice (*n* = 8,984) based on gene expression profiles. The cell population marked by three colors according to the species. **b** t-SNE visualization of 38,780 cells grouped by expression similarity and colored by cluster. **c** Histogram showing the percentage of all cell types in ileum tissue from mice, pig and human. **d** Heatmap showing the differential expression of marker genes in epithelial, stromal, and immune cell subsets from human, pig and mice data. **e** Representative conserved signature genes in each cell subset from mice, pig, and human ileum. The darkness of red color corresponds to average expression level of genes. Dot size indicates the percentage of cells expressing the corresponding genes. **f** Quantity of epithelial, stromal, and immune cell subsets at different cell cycle from ileum of mice, pig, and human. **g** Pearson correlations between each pair of single cells from mice, pig, and human datasets based on their gene expression profiles. Black-dotted boxes showing the correlations of single-cell pairs between human and mice were relatively low and unstable

After obtaining a comprehensive atlas of single cell transcriptional states in ileum from piglets, we performed an across-species, comparative analysis by aligning the piglet data to previously obtained mice and human intestinal datasets, enabling to distinguish between both conservation or divergence of cellular architecture and molecular characteristic across species. Homologous gene data (comparison tool: BioMart of the Ensembl) were re-analyzed uniformly to merge the cell data across three species. The clustering arrangement of the cells from the different species was broadly overlapping according to major cell-type categories rather than species (Fig. 4a, b). Transcriptomic cluster types were relatively conserved across species, as each selected cluster contained cells from all species (Fig. 4c). Among these cells, a total of 12 clusters were identified according to the specific expression of marker genes (Fig. 4d), mainly including ProgC, EC, PC, GC (sum to 50.30%), immune cells (> 40%), and endothelial cells (3.4%). We separately examined expression patterns of the cell-type-specific marker genes from humans, mice, and piglets to compare conserved molecular signatures across species. The level of retained cellular property was quantified by the shared marker gene expression programs in mammalian ileum epithelial and immune cell subtypes, suggesting that ileum carries out similar overall functions throughout three species (Fig. 4e). However, the similar expressions of conserved cell-type specific genes were robustly observed in humans and piglets but not in mice. For example, *FABP2* and *APOA1* in ECs, *DEFA* and *ANG* in PCs, and *SPKIN4* and *TFF3* in GCs were consistently expressed in human and pig samples but exhibited very low expression in mice. Notably, multiple cell markers in T and B cells shared by pigs and humans showed a distinct expression pattern in mice. This result suggests that transcriptomic immune cell signatures of humans are not fully conserved in mice but more closely resemble pigs. Cell-cycle analysis revealed that the proportion of annotated cell-cycle status in each cluster was similar across species (Fig. 4f). The distributions of different cell cycle phases in major cell types were again similar between humans and pigs, such as ProgC, EC, PC, and partial immune cells. In order to compare cellular transcriptional states across-species, we computed pairwise Pearson correlations between humans, mice, and pigs based on the corresponding gene expression profiles. We found that the correlations between mice and humans were appreciably reduced in the same cluster of cells, while they were largely maintained in humans and pigs (Fig. 4g). These results suggest that humans share high transcriptomic similarity with pigs, whereas the evolutionary divergence of gene expression patterns between humans and mice are indeed greater.

### Inflamed ileum of piglet post weaning showed mitochondria damage in epithelial cells

**Fig. 5:**
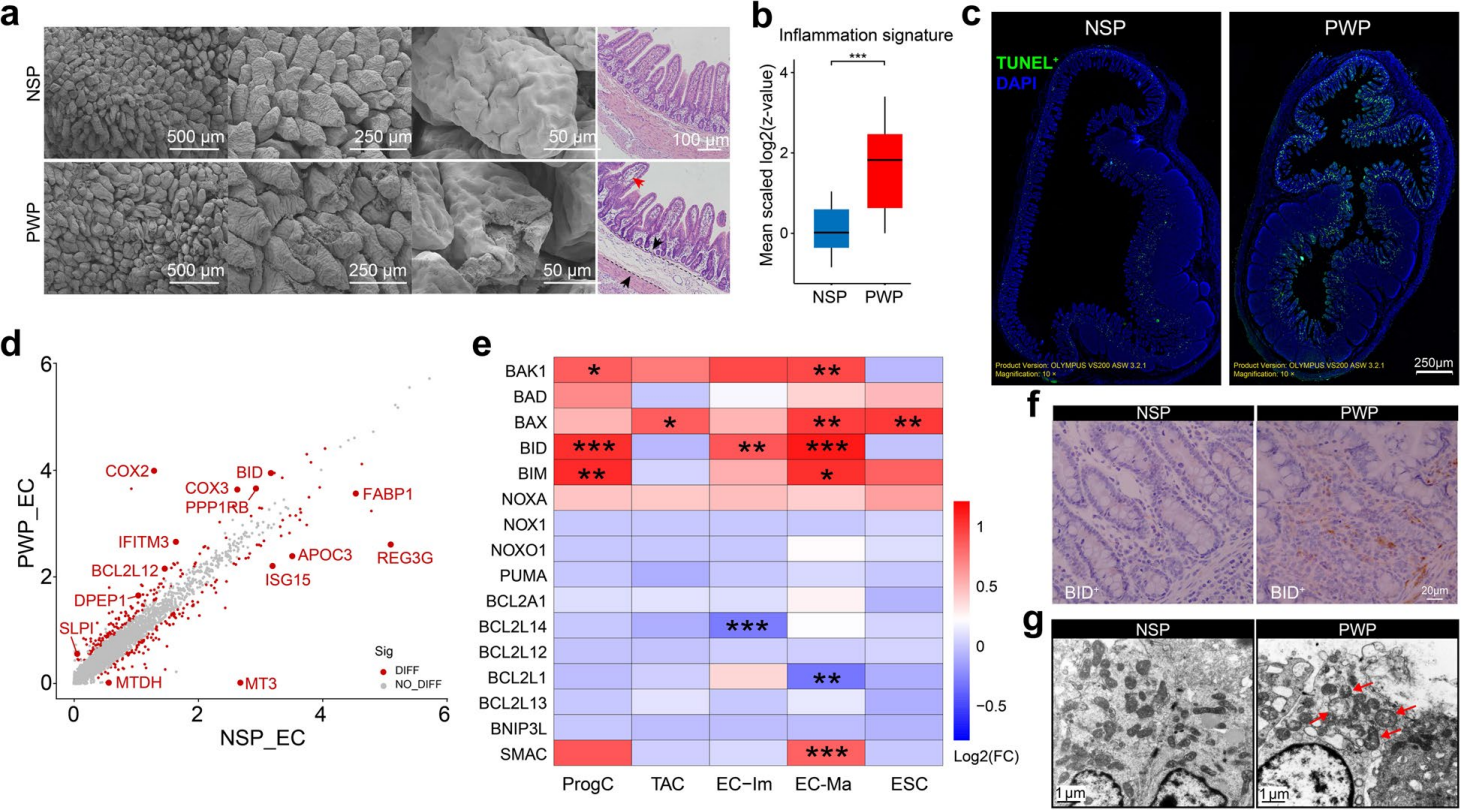
Weaning induced ileum inflammation and mitochondrial damage in epithelial cells. **a** Scanning electron microscope of the ileum tissue from NSP and PWP (left panel); H&E staining (right panel). **b** Boxplots depicting mean gene expression of inflammatory signatures from NSPs and PWPs, ***p<0.001; error bars: SEM; Wilcoxon test. **c** TUNEL staining in ileum tissues from NSP and PWP. The nuclei of apoptotic cells are stained with green. Scale bars, 500 μm. **d** Scatterplots showing differential gene expression in enterocytes between PWPs and NSPs. Genes with *p* value<0.05 and log2 (FC)>0.25 are shown. **e** Heatmap shows the log2 (FC) in gene expression of BCL2, BAX, and BH3-only family genes between NSPs and PWPs. *p<0.05, **p<0.01, ***p<0.001, Wilcoxon test. **f** IHC images of ileum tissue from NSPs and PWPs stained for BID (*n*=6). Scale bars, 20 μm. **g** TEM analysis shows mitochondrial ultrastructure of enterocytes (*n*=6). Red arrows indicate damaged mitochondria with disrupted respiratory cristae. Scale bars, 1 μm

The process of weaning can induce complex intestinal disturbances, characterized by morphological perturbations or gut inflammation [11]. According to the intestinal morphology, the ileum of PWPs displayed severe inflammation in the villus, mesenchymal swelling, and epithelial stratification (Fig. 5a). The expression of a specific inflammation-associated gene set calculated from the scRNA-seq profiles verified a shift in mucosal immune status (Fig. 5b), which is consistent with a previous report of the intestinal inflammatory response after weaning [8]. Moreover, IF staining indicated a significantly higher number of TUNEL^+^ labeling apoptotic cells in the PWP group than in the NSP group (Fig. 5c and Additional file 14: Fig. S7a). The expression of cytochrome family genes (*COX2*, *COX3*, and *CYTB*) was observed in a wide variety of epithelial cells, especially those from the PWP group (Fig. 5d and Additional file 14: Fig. S7b). We speculated that the mitochondrial membrane was damaged and caused cytochrome release right after the weaning stress was perceived. We further observed the expression of anti-apoptotic and pro-apoptotic genes relating to mitochondrial membrane function across all epithelial cells, such as *BCL2*, *BAX*, and *BH3*-only family genes. Abnormal expressions of these genes could lead to destabilization of the mitochondrial membrane structure and to the release of apoptotic factors, including cytochrome and *SMAC* [47]. Functionally, DEGs from the two groups were performed by GO enrichment analysis, highlighting major processes including signals of mitochondrial function and inner membrane molecules (Additional file 14: Fig. S7c). Among these genes, the pro-apoptotic members *BID* (with largest expression range across all epithelia cell types), *BIM*, and *BAK1* were upregulated in the PWP group compared to the NSP group (Fig. 5e), suggesting a possible damage of mitochondrial membranes in the PWP group. We performed immunohistochemical (IHC) staining against *BID*^+^ to corroborate the scRNA-seq data (Fig. 5f). Ultrastructural analysis revealed that mitochondria were engulfed by vacuoles and had swelling and disruption of mitochondrial cristae membranes in the PWP samples (Fig. 5g), well in line with the hypothesis of mitochondrial membrane disruption.

### Distinct states of intercellular signaling networks between NSP and PWP groups

**Fig. 6:**
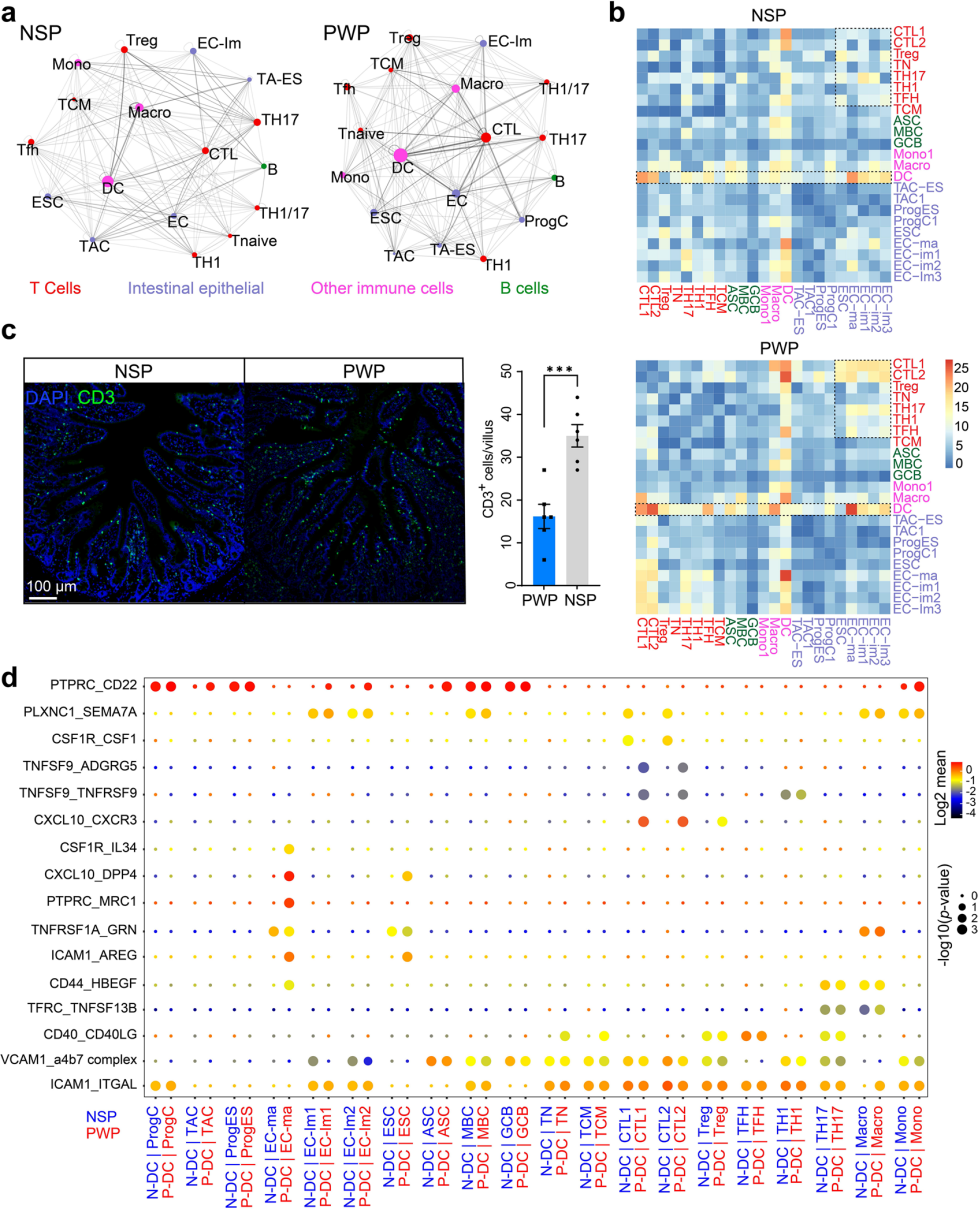
Intercellular communication networks among cell types in the ileum of NSPs and PWPs. **a** Network graph showing interactions in NSP (right panel) and PWP (left panel) between two cell types coded in colored circles. Networks depicting clusters as nodes and interactions as edges. The sizes of each cluster type are proportional to the total interaction count in each cell type, and edge thickness is proportional to interaction number between connecting types. See Additional file 15: Table S8 for detailed information. **b** Heatmap showing the number of ligand–receptor pairs across cell subgroups from NSPs (right panel) and PWPs (left panel), predicted by CellphoneDB. Cell types are grouped by broad lineages (intestinal epithelial cells, T cells, B cells, or other immune cells). **c** The recruitment of CD3^+^ T cells, identified by immunofluorescence (IF) in the ileum villus. Top: representative IF image showing CD3^+^ (green) expression in NSPs and PWPs. The numbers of CD3^+^ T cells significantly increased in PWPs compared with NSPs (*n*=6). Scale bar represents 100 μm. Bottom: the number of CD3^+^ cells in the field of each villus (***p<0.001, Student’s t-test; error bars: SEM). **d** Bubble plots showing significant ligand/receptor pairs between dendritic cells (DCs) and another cell types in NSPs and PWPs. Some selected DC–enterocyte (EC) and DC–cytotoxic T lymphocyte (CTL) interactions were enriched in PWPs but absent in NSPs. Point sizes indicate the permutation *p* value (CellPhoneDB). Colors reflect expression levels of ligands and receptors

To perform dynamic modeling of the crosstalk among ECs and immune cells in NSPs and PWPs, we assessed cell–cell communication by examining the expression of ligand and receptor molecules [48]. Based on the clustering results, we mapped landscapes of interaction networks between the epithelial and immune cell subtypes. We identified 1193 significant interactions among 715 putative ligand–receptor interacting pairs across 32 cell types (Additional file 15: Table S8). Extensive interactions were identified among DCs, CTLs, and ECs (center of network diagram) in both NSPs and PWPs. Higher frequencies of the corresponding cell–cell communications were detected in PWPs than in NSPs (Fig. 6a and Additional file 15: Table S8).

We predicted that the DC populations harboring the maximum numbers of cell-type-specific crosstalk with other neighboring cells (up to 356 interaction counts in NSPs vs. 431 in PWPs) served as hubs in the cell–cell crosstalk in both NSPs and PWPs (Fig. 6b). Altered cellular communication was detected in PWPs compared with NSPs. In PWPs, the numbers of predicted interactions between ECs and multiple T subsets were increased, especially between CTL and Th17. Interactions between DCs and CTLs increased, and DC–Th17 interactions were increased slightly in PWPs compared to NSPs. Our results indicate an altered spatial distribution of *CD103*^+^ DCs within the villus and lamina propria in PWPs relative to NSPs, with more DCs migrating into the ileum villus in PWPs (Additional file 16: Fig. S8a), supporting intensive cellular interactions between DCs and ECs, as indicated by the scRNA-seq data. We subsequently performed IF staining to provide evidence that CD3^+^ T cells, irrespective of their type specificities, significantly migrated into villus tissue, implying unique immune cell recruitment and modulation, as indicated by the identified population structures in PWPs (Fig. 6c). We postulated that DCs were remodeled in the piglet’s small intestines during weaning to obtain immunomodulatory functions and to promote antigen uptake and T cell priming.

To further examine chemoattraction-mediated intercellular communication, we assessed the specificity of ligand–receptor pairs, which displayed significant cell state differences in our scRNA-seq dataset. We validated that DCs were more likely to interact with CTLs in both groups. However, some pairs were unique to NSPs or PWPs. For instance, the ligand–receptor pairs of *CXCL10–CXCR3*, *TNFSF9–ADGRG5*, and *TNFSF9–TNFRSF9* were significantly enriched in PWPs (Fig. 6d), which was consistent with the results of universal gene activation in PWPs (such as *CXCL10*, *ADGRG5*, *TNFSF9*; Additional file 17: Fig. S9a, b). By contrast, the *CSF1R–CSF1* and *PLXNC1–SEMA7A* interaction pairs were significantly enriched between DCs and CTLs in NSPs. These findings suggest that resident DCs have the capacity to recruit CTLs via particular ligand–receptor signals and that the specificity of cytokine interactions (*TNFSF* and *CXCL10*) might contribute to mucosal inflammation in PWPs [16, 44]. We observed that the *CXCL10–DPP4*, *PTPRC–MRC1*, *CSF1R–IL34*, and *CD44–HBEGF* interactions increased between DCs and ECs in PWPs but not in NSPs (Fig. 6d). By comparing the expression of ligands and receptors in DCs and ECs, we found that *PTPRC*, *MRC1*, and *IL34* displayed high expression levels in ECs in PWPs, and *DPP4* showed high expression in both NSPs and PWPs (Additional file 17: Fig. S9c, d). Similarly, we tested ligand–receptor pairs between ECs and other cell subsets and explored DEGs in each cluster (Additional file 16: Fig. S8b and Additional file 17: Fig. S9a). The results pointed to interactions between the intestinal epithelial chemokine *CXCL10* and the Th17-expressed receptor (*DPP4*) or the CTL-expressed receptor (*CXCR3*) that were significantly enriched in PWPs compared to NSPs.

### Th17 cells displayed inflammation-associated and plasticity signatures in PWPs compared with NSPs

**Fig. 7:**
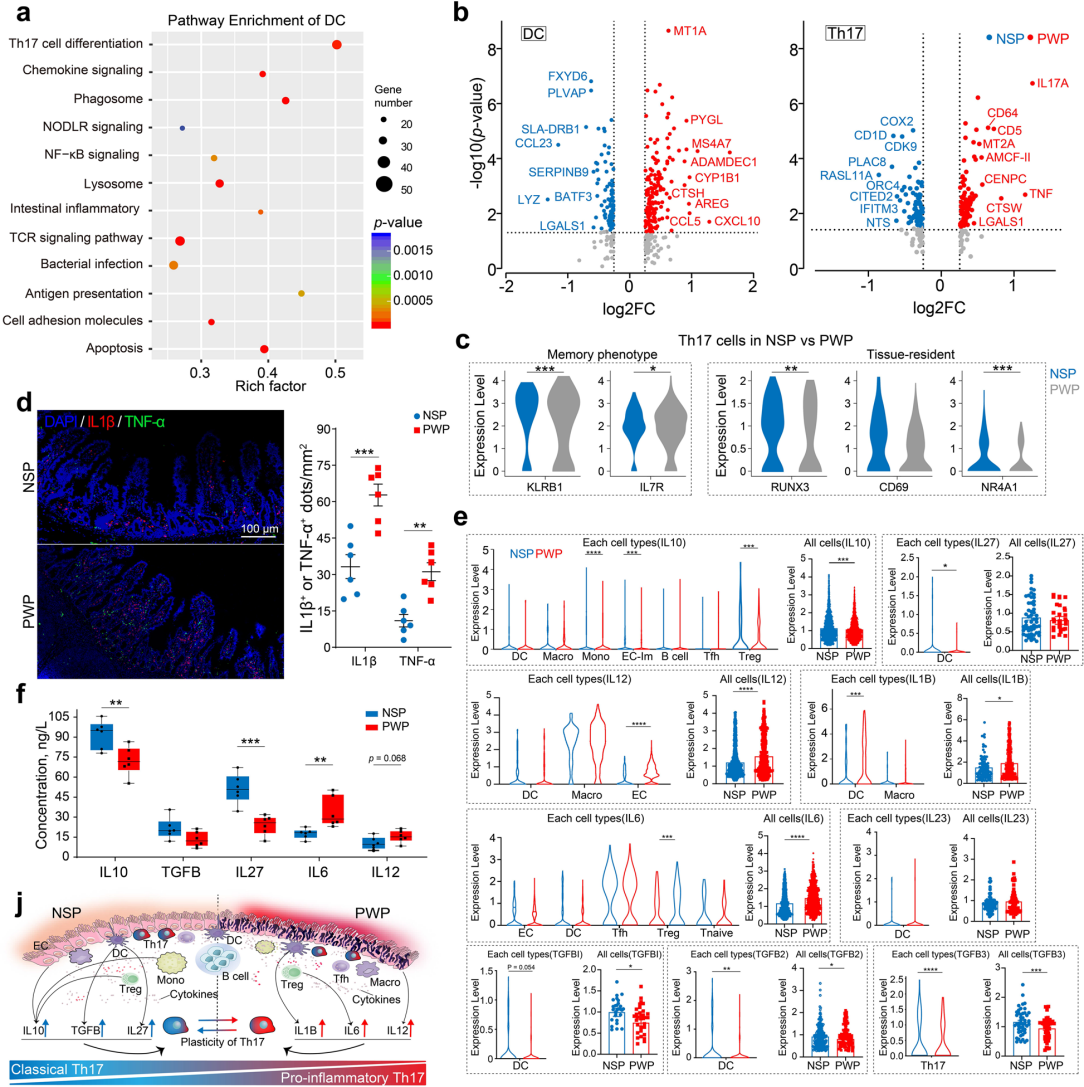
Cytokine-regulated plasticity of Th17 cells toward a pro-inflammation signature in the piglet ileum. **a** GO analysis of dendritic cell (DC) cluster-derived differentially expressed genes. Selected GO terms with Benjamini-Hochberg-corrected *p* values<0.05 (one-sided Fisher’s exact test). **b** Volcano plots for differentially expressed genes between DCs (left) and Th17 cells (right) in PWPs relative to NSPs. Hurdle model likelihood ratio test for differential expression, Benjamini–Hochberg multiple testing correction. All genes given in Additional file 18: Table S9. **c** IF staining showing the increased abundance of *IL1β* (red) and *TNF*-α (green) in PWPs compared with NSPs (*n*=6). Left: images of *IL1β* (red) and *TNF*-α (green) antibody staining; scale bars, 100 μm. Right: number of colored dots in the field of view (mm^2^) (*n*=6); **p<0.01, ***p<0.001; error bars: SEM; Student’s *t*-test. **d** Violin plot showing the gene expression levels relating to memory and tissue-resident phenotypes in Th17 cells from NSPs and PWPs. *p<0.05, **p<0.01, ***p<0.001, Wilcoxon test. **e** Violin plot comparing cytokine gene expression levels in T cell subpopulations. The specific cell types that express target cytokines are given in Additional file 19: Fig. S10f. *p<0.05, **p<0.01, ***p<0.001, Wilcoxon test. **f** ELISA showing changes in porcine cytokine production. *p<0.05, mean ± SEM (*n*=6), Student’s *t*-test. **j** Schematic drawing showing Th17 plasticity-regulated changes in cytokine environment

Analyses of distinct T cell subsets (Fig. 3j) and cell–cell interaction frequencies (Fig. 6b) proposed that intestinal DCs may generate signals to promote T cell differentiation. Functional enrichment analysis indicated that the genes associated with Th17 cell differentiation, chemokine signaling, and antigen presentation were enriched in the DC cluster (Fig. 7a). We further identified DEGs in DCs between NSPs and PWPs and performed functional enrichment analysis. We observed that multiple genes relating to Th17 differentiation increased in DCs obtained from NSPs, consistent with the increased fraction of Th17 cells observed in NSPs versus PWPs (Fig. 7b left and Additional file 18: Table S9). However, genes associated with inflammation, apoptosis, and *TNF*-mediated signaling were enriched in DCs from PWPs (Additional file 19: Fig. S10a). Similarly, GO functional enrichment analysis showed that genes enriched in the *TNF* signaling pathway, intestinal inflammation, and cytokine–receptor interactions were highly expressed in Th17 cells (Additional file 19: Fig. S10b). Comparison of Th17 cells between NSPs and PWPs indicated distinct expression of multiple genes (Fig. 7b right) relating to *TNF* expression, inflammatory responses, T cell co-stimulation, and apoptotic signaling pathway activation (Additional file 18: Table S9). The expression of memory phenotype genes, such as *KLRB1* and *IL7R*, was upregulated in Th17 cells from PWP samples, while the expression of tissue resident genes involved in *RUNX3*, *NR4A1*, and *CD69* was reduced (Fig. 7c). Our results demonstrate that Th17 cells were activated after developing from Tn cells in the PWP group. Notably, the expression of risk genes for inflammation, such as *TNF*, *IL7A*, and *CD64*, was significantly increased in the Th17 subset in PWPs than in NSPs. *CD64*, an IgG high-affinity receptor, participates in *IL1β* processing and migration of pathogenic Th17 cell [49].

We postulated that overexpression of *TNF* in Th17 cells might be associated with inflammation in the ileum. Ligand–receptor analysis showed the interaction between Th17 cells and intestinal epithelial cells or immune cells via crosstalk between *TNF* and multiple corresponding receptors in NSPs and PWPs (Additional file 19: Fig. S10c). In addition to the expression of *TNF*, we compared the transcription profiles of the corresponding receptor genes in other cell types. Indeed, we observed the elevated expression of receptor genes, which were widely distributed in various PWP cell types, suggesting intense interactions (Additional file 19: Fig. S10d, e). To assess expression differences and the origination of *TNF*, we performed IF staining and found significantly increased *TNF*-α protein expression in the ileum mucosae of PWPs compared with NSPs (Fig. 7d). Th17 cells were the dominant source of *TNF* expression in PWP samples relative to the amount of *TNF* expressed by other T cell subsets. *TNF* expression was significantly higher in Th17 cells in PWPs than in NSPs (Additional file 19: Fig. S10d). These data suggest that Th17 cells have phenotypic flexibility (defined as functional plasticity), in line with previous findings [21], and that Th17 cells acquire pro-inflammatory properties in response to weaning.

Th17 cells and their developmental plasticity were affected by the local cytokine milieu [20]. To determine which cytokine specifically may mediate Th17 cell signaling during weaning, we first performed a comprehensive screening of the overall transcription profile to identify which cell types expressed the target cytokines at high levels. For example, our data revealed a significant upregulation of *IL12* expression in DCs, macrophages, and ECs (Additional file 19: Fig. S10f). We calculated the expression level differences in each identified cell type between PWPs and NSPs. Next, we separately pooled each cell type expressing the corresponding cytokines to determine the overall significant differences between NSPs and PWPs (Fig. 7e). Finally, to confirm differences in the cytokine environments between NSPs and PWPs, we evaluated concentration changes in the specific cytokines between the two groups by enzyme-linked immunosorbent assay (ELISA; Fig. 7f) or IF staining (Fig. 7d for *IL1β*) to validate the differences inferred by scRNA-seq data. Collectively, we identified multiple cytokines that are differentially released by various cell types in response to weaning. IL10 (derived from Treg, monocytes, and EC cells), IL27 (DC-derived), and TGFB (DC-derived) were representative anti-inflammatory cytokines enriched in NSPs, whereas cytokines with pro-inflammatory properties, such as *IL1β*, *IL6*, and *IL12* were highly released in PWPs (Fig. 7j). These differences in expression of the cytokine milieu may be more closely associated with pathogenicity than the differentiation of Th17 cells, which is consistent with a previous study [50].

### CD8^+^ CTLs, with enhanced inflammatory characteristics, are enriched in ileum tissue from PWPs

**Fig. 8:**
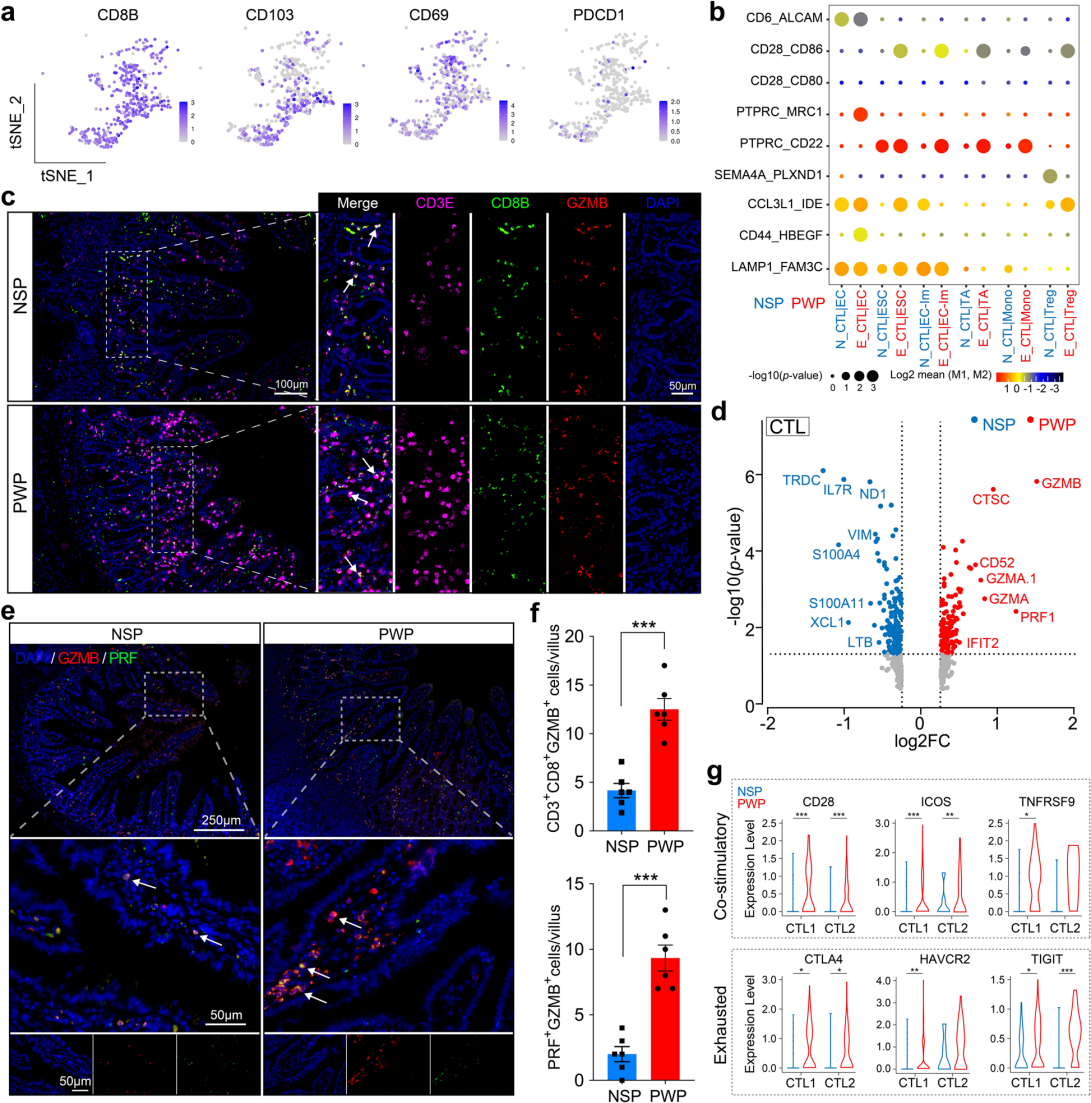
Pathogenic effector CTLs enriched in PWPs compared to NSPs. **a** t-SNE plot of *CD8B*, *CD103*, *CD69*, and *PDCD1* expression levels on CTLs. **b** Bubble plots showing significant ligand/receptor pairs between CTLs and other cell types. Point sizes indicate permutation *p* values (CellPhoneDB). **c** IF staining showing the infiltration of *CD3*^+^ *CD8*^+^ *GZMB*^+^ CTLs in PWPs. 5× magnification (right). Scale bars, 100 μm. **d** Volcano plots of differentially expressed genes in CTLs obtained from PWP relative to NSP. Hurdle model likelihood ratio test for differential expression, Benjamini–Hochberg multiple testing correction. All genes are given in Additional file 18: Table S9. **e** IF staining showing the co-expression of *PRF* and *GZMB* in CTLs. Scale bars, 100 μm. **f** Cell densities (number of cells/villus) of *CD3*^*+*^ *CD8*^*+*^ *GZMB*^*+*^ CTLs and *GZMB*^*+*^ *PRF*^*+*^ CTLs of NSPs and PWPs, according to Fig. 6c and e. ***p<0.001. Student’s t-test. Error bars: SEM (*n*=6). **g** Violin plot showing the expression of costimulatory and exhausted gene sets in CTLs of NSPs and PWPs. Wilcoxon test. *p<0.05, **p<0.01, ***p<0.001

Although we recognized the pathogenic potential of Th17 cells, these are not characteristic cytotoxic T cell types in the ileum during inflammation. Our new data demonstrate preferential accumulation of CD8^+^ CTLs in PWPs (Fig. 3j). Both *CD8*^+^ CTL subsets simultaneously express effector and cytotoxic genes, including *CD8B*, *GZMB*, and *PRF1* (Fig. 8a and Additional file 20: Fig. S11a). In addition to similar transcriptional characteristics, one of the *CD8*^+^ CTL cell types expressed high levels of transitioning and proliferating genes, including *P*C*NA*, *MKI67*, and *TK1* (Additional file 10: Table S7), implying their intermediate or dynamic cytotoxic properties. The slight expression of the T cell exhaustion marker (*PDCD1*) and DEG pathway analysis confirmed the high potential to mediate intestinal inflammatory responses in *CD8*^+^ CTLs (Fig. 8a and Additional file 20: Fig. S11b). In addition, CD8^+^ CTLs highly expressed the lymphocyte-residence genes *CD69* and *CD103*, suggesting that these cells were equipped with an anchoring ability in tissue [51] (Fig. 8a). To validate the potential interconnections, we estimated potential cellular crosstalk strength pertaining to *CD8*^+^ CTLs by examining differences in ligand–receptor pairs. Our data propose the existence of specific interactions between *CD8*^+^ CTLs and other cell types via *CD28–CD86* and *PTPRC–CD22* pairs in PWPs but not in NSPs (Fig. 8b). Moreover, *PTPRC* was highly expressed in *CD8*^+^ CTLs in the PWP group while their corresponding ligands were induced in multiple intestinal epithelial subpopulations (Additional file 20: Fig. S11a). Next, we examined differential transcriptomic profiles in *CD8*^+^ CTLs between NSPs and PWPs and identified 275 DEGs (adjusted *p* value<0.05, Additional file 18: Table S9). By contrast, several established cytotoxic effector genes, including *GZMB*, *PRF1*, and *CTSC* (top-regulated genes), were upregulated in PWPs, whereas *TRDC*, *IL7R*, and *LTB* were abundant in NSPs (Fig. 8d). Cathepsin C (encode by *CTSC*) was characterized as an activator of the pro-inflammatory granule-associated serine protease, which was involved in *GZMB* activation [52]. We also assessed gene expression of costimulatory and exhausted T cell phenotypes. Intriguingly, *CD8*^+^ CTL subsets were increased in PWP as being the expression of co-stimulatory (*CD28*, *ICOS*, and *TNFRSF9*) and exhausted molecules (*CTLA4*, *HAVCR2*, and *TIGIT*; Fig. 8g), which may point to a hyper-activation cytotoxic T cell state. Across all PWP samples, *CD8*^+^ CTL cells highly expressed pathogenic effectors consistently throughout the mucosa. Consequently, an advanced immunosuppressive response occurred, once they had been locally enriched. We also observed a high expression of *SERPINB9* in DCs from NSP group (Fig. 7b, left). Protease inhibitor-9 (encoded by *SERPINB9*), a potential cellular inhibitor of *GZMB* [53], supported the above results.

To verify the physical juxtaposition and enrichment of *CD8*^+^ CTLs in PWP samples, we performed multiplexed IF staining for *CD3E*, *CD8B*, and *GZMB*. We observed that *CD3E*^*+*^ *CD8B*^*+*^ *GZMB*^*+*^ CTL cells resided near intestinal epithelial cells and that high levels of lymphocytic infiltration existed in ileum tissues from the PWP group (Fig. 8c, f up). *PRF1* promotes the internalization of *GZMB* into targeted cells, causing the initiation of apoptosis [54]. The double IF staining of *GZMB* and *PRF* confirmed that *CD8*^+^ CTLs concomitantly released perforin and granzymes, which were both overexpressed in PWPs (Fig. 8e, f down). Thus, the activation of a cytotoxic program associated with *CD8*^+^ CTLs may have aggravated ileum tissue damage in our samples. We calculated the expression level of genes relating to caspase-dependent pathways. However, no significant differences were observed in caspase target genes among epithelial cell types from the PWP and NSP groups (Additional file 20: Fig. S11c). Thus, we speculated that there is another *GZMB*-induced, necrotic cell death mechanism through a caspase-independent pathway, confirming a previous study [55]. Together, our data demonstrate that enrichment of intestinal *CD8*^+^ CTLs triggers cytotoxicity (*GZMB* and *PRF1*) and might contribute to mitochondrial dysfunction and might give rise to further inflammation process in the ileum.

## Discussion

Facilitated by leveraging scRNA-seq, we characterized gene expression profiles of cell subpopulations and identified differences in cellular components, differentiation hierarchies, cell–cell interactions of structural and immune cells in ileum tissue from NSP and PWP. Comparing our new data with similar ones from human and mice scRNA-seq datasets revealed a well-conserved cellular architecture and transcriptional program in humans and pigs.

We characterized three ESC subtypes (GC, EEC, and B/OC) according to the expression of hallmark genes, suggesting similar intestinal secretory cell lineages between pigs and humans [23, 34]. IF assays demonstrated that analogous subgroups of secretory cells exist across the ileum tissue of all piglets, based on the subtypes and anatomical locations of ESCs. Computationally derived differential trajectories revealed that GC, EEC, and B/OC moved along a single path that is separate from ECs, indicating that secretory cell subtypes exhibit an overlapping gene expression profile, as has been reported from humans [34]. During cell fate determination, the suppression or upregulation of fate-specific gene transcription plays an important role in the functional maturation of each cell type (Fig. 2f and Additional file 4: Fig. S2d). For example, we observed that numerous genes encode structural constituents of ribosomal proteins, showing dynamic expression during differentiation, in agreement with previous findings [36, 56].

The transition period during weaning is associated with a critical perturbation in the intestinal immune system [57]. Since we might have captured distinct immune cells asynchronously transitioning from one transcriptomic state to the next, we performed pseudotime analysis to capture a linear trajectory of T and B cell subpopulations, which were consistent with previous findings [39, 41]. Importantly, the combination of single-cell trajectories and cell status hallmark gene expression data allowed for an insight into immune cell activation status. The unique transcriptional states of T cells were characterized by expanding cytotoxicity in the PWP group, enabling us to better explore the link between T cell subtype pathogenicity and tissue damage [8, 58]. Notably, the primary differences identified in the T cell composition were the exclusion of Th17 cells and the preferential enrichment of CTLs in PWPs compared to NSPs.

In both NSPs and PWPs, DCs acted as central nodes in the landscape of cell–cell interaction networks, while some specific intercellular crosstalk was altered in PWPs. Chemokine gene *CXCL10* was reported to orchestrate expansion of DCs into the villus-crypt region, while mediating antigen presentation and primary cytotoxic T cell immunity [59]. Therefore, DCs exert an important role in recruiting immune cells into inflammation sites. DEG profiling could identify heterogeneity and functional changes in DCs between NSPs and PWPs. We observed that DCs from PWP samples showed pro-inflammatory signals and T cell activation, similar to previous findings [42, 60], suggesting that T cell subpopulations should receive additional attention in future studies.

By identifying differences in cell proportions and cellular states based on transcriptional data and cellular crosstalk data, we identified Th17 and CTL cells as potentially important players in the mediation of intestinal inflammation during weaning in piglets. Th17 cells possess remarkable pro-inflammatory transcriptional characteristics, including the significantly elevated expression of *IL17A* and *TNF* in PWPs compared with NSP, suggesting that Th17 cells are very plastic under certain conditions [50]. Th17 cells were found to be major *TNF*-α-producing cells during weaning, and *TNF* mediates cell-cell communication with other intestinal epithelial cells. This suggests that Th17 cells may be responsible for pathogenicity and may contribute to tissue damage [51, 61]. Our analyses uncover an environmental immune adaptation of Th17 cells, which orient themselves towards a pro-inflammatory or classical immunoregulatory fate depending on the local cytokine milieu. In the PWP group, Th17 cells shifted towards an inflammatory state and were associated with increased abundance of cytokines with pro-inflammatory properties including *IL1β*, *IL6*, and *IL12* [62, 63]. CTLs, representing a unique population among T cell subsets, highly expressed genes with pro-inflammatory and cytotoxic effector features including inflammatory cytokines, perforin (*PRF*), and cytolytic granules (*GZMB*). IF staining analysis confirmed the proliferation and infiltration of *CD8B*^*+*^ *GZMB*^*+*^ CTLs in the ileum tissue obtained from the PWP group, while they were rarely detected in NSP expectedly. Co-stimulatory and exhaustion assays in CTLs suggest efficient activation of a cytotoxic process in the PWP. CTLs showed stronger interactions with various intestinal epithelial cells in PWPs than in NSPs, suggesting that intestinal inflammation might have been exacerbated in PWPs relative to NSPs. Therefore, we compared DEGs in various structural cells and identified many genes relating to mitochondrial dysfunction as being highly expressed in PWPs, but the process was not associated with caspase activation. We speculate that the *GZMB* accumulate, secreted by CTLs, and enter target intestinal epithelial cells by perforin-mediated internalization, through which CTLs give rise to intestinal inflammation [54]. The pro-apoptotic factors *BAX* and *BH3*-only family genes, such as *BID*, *BIM*, and *BAK1*, can be directly activated by granzyme B through a caspase-independent pathway [55], resulting in their translocation into the mitochondria while inducing apoptosis by binding and antagonizing anti-apoptotic *BCL2* family genes.

## Conclusions

Overall, this study provided a single-cell transcriptional landscape atlas of ileum tissue of piglet. Integrated cross-species comparisons reveal similar single-cell transcriptomic profiles of ileum tissue in humans and pigs. We investigated differences in cell proportions, gene expression patterns, and cell–cell signaling between NSPs and PWPs. Finally, we identified altered Th17 plasticity regulated by the cytokine population in PWPs, and the enrichment of *GZMB*^+^ CTLs was associated with extensive mitochondrial dysfunction, which promoted inflammation in the ileum of PWP. Thus, our new atlas provides the molecular foundation for investigating cell identities, function, and intercellular crosstalk in ileum mucosa of piglets. Pigs may serve as insightful animal model to better understand weaning-induced gastrointestinal diseases.

## Methods

### Animal care and sample processing

All procedures were conducted in accordance with the treatment protocols outlined for the Center of Laboratory Animals at Zhejiang University and the Animal Care and Use Committee. We selected three litters with 12 piglets in each litter (sow: Landrace × Yorkshire). After 21 days of lactation (from birth to weaning stage), the piglets from same litter were divided into two groups (*n* = 6): the PWP was transferred to a nursery house while getting isolated from the mother sow, whereas NSPs were still nursed by mother sow. The PWP group was provided with ad libitum access to solid food pellets. At an age of 26 days, piglets were euthanized with an intravenous injection of sodium pentobarbital solution (25 mg/kg body weight). Mid-segments of the ileum were obtained immediately and processed for single-cell suspension. Adjacent tissues to the ileum were fixed with 2.5% glutaraldehyde for morphological observation. Fresh ileum segments were stored in buffered formalin solution before getting embedded in paraffin for morphological measurements (H&E) and IF staining assays. Mucosal scrapings from the ileum were collected with a microscope slide, immediately placed into −80 °C before the running of ELISA.

### Cell suspensions for single-cell sequencing

The whole thickness of the ileum tissue was immediately transferred into a sterile, RNase-free culture dish containing cold 1× PBS (no calcium and magnesium), where it was sliced into roughly 0.4-mm^2^ pieces. The tissue was rinsed with 1× PBS to clear off fat layers and blood. The tissue was then brought into 5-ml digestion solution (2.5 mg/ml papain, 0.4% collagenase IV5, 140 units/ml DNase I) for 15 min at 37 °C. All enzymatic reactions were stopped with fetal bovine serum (FBS) (50% v/v), then pipetted 6 times. The resulting cell suspension from filtration with an 80-μm restrainer was centrifuged at 300×*g* for 5 min at 4 °C. The pellets were mixed with 100-μl resuspension buffer (0.04% bovine serum albumin [BSA]) and 900-μl red blood cell lysis buffer (MACS, 10×) and incubated for 5 min at 37 °C. The suspension was transferred into 50 μl 1× PBS (0.04% BSA) after removing damaged cells using a Miltenyi® Dead Cell Removal Kit (MACS, 10× Genomics). The overall cell counts (800–1200 cells/μl) and viability (above 90%) were detected with a hemocytometer/Countess II Automated Cell Counter (Thermos Fisher).

### 10× library preparation for sequencing

The single cell suspension was processed into 10x Chromium single-cell platform to isolate an appropriate number of single cells as described in the manufacturer’s protocol for a Chromium Single Cell 3’ V3 assay (10x Genomics, USA). Complementary DNA amplification and library construction were done according to the manufacturer’s recommendations. Libraries were subjected to the Illumina NovaSeq 6000 sequencing platform (paired-end multiplexing run, 150 bp) to achieve 52,311 reads per cell (average of each libraries).

### Quality control and bioinformatics analyses of scRNA-seq data

The sequencing output was demultiplexed by Illumina bcl2fastq system (V2.19.1) and converted into the FASTQ format. Cell distinguishing barcode information and UMI demultiplexing were processed with the Cell Ranger pipeline using recommended parameters, and reads were aligned with the Ensembl genome *Sus scrofa* reference genome annotation. A total of 50,025 single cells (six samples) were captured using 10× Genomics Chromium Single Cell 3’ Solution. The filtered counting matrix (filtering non-cell associated barcodes) was loaded into the package Seurat (R environment, version 3.1.1) for dimensional reduction, clustering, and analysis of the merged data [64]. Doublet scoring was analyzed using the R implementation of scrublet. Overall, 42,149 cells passed the quality control threshold, and the following were eliminated: genes expressed in fewer than 1 cell, genes per cell ≤ 500 or ≥ 5800, UMI counts amounting to less than 50,000, and cells with a percentage of mitochondrial RNA-derived gene-expression < 20%.

We further performed dimensionality reduction for all 42,149 cells using the Seurat R package and visualized the results as follows: the expression value of each gene was calculated (logNormalize, “Normalization” function), and RunPCA algorithm was performed using the normalized gene expression lists. The top 20 PCs were used to calculate t-SNE for the dataset. Cells were clustered using the shared nearest neighbor (SNN) graph-based method. Differentially expressed genes for each cluster were analyzed using the “bimod” (Likelihood ratio test) setting, with default parameters, via the FindAllMarkers function. The clusters were classified based on canonical genes with high expression level. Initial clusters were identified as intestinal epithelium cells, secretory cells, immune cells, and mesenchymal cells according to the average gene expression for each cluster (Additional file 1: Table S1). A second clustering step was performed for T cells (Clusters 11, 18, and 19), B cells (Clusters 1, 7, 13, and 15), and secretory cells (Cluster 20), based on the top 10 PCs, with specific resolution setting. Similar clustering results were obtained with the Seurat R package (version 3.1.1). The t-SNE visualization was performed using the R packages RunTSNE and DimPlot, with default settings.

### Cell-cycle scoring

We carried out cell-cycle stage annotation for each cell by using Cell Cycle Scoring package in Seurat. First, we calculated a score for each cell based on the geometric mean expression of gene sets reflecting the G2/M phase (51 marker genes) and the S phase (39 marker genes) (Additional file 21: Table S10). Cells expressing neither G2/M phase markers nor S phase markers were assigned to the G1 phase. Secondly, we used a centering and normalizing method to normalize the phase score across all the phases within each cell. Then, we stored the G2/M and S scores in the object meta data and visualized the classification of each cell in each cell-cycle phase.

### Pseudotime analysis

Differential expression pattern along the cell state transition trajectory was calculated using the Monocle 2 (version 2.8.0) [65]. Pseudotime analysis was performed for ESCs and T and B cell subtypes using the general pipeline (http://cole-trapnell-lab.github.io/monocle-release/docs/). We used the top 100 DEGs in ESC and B cell subtypes (identified by Seurat) to perform dimensionality reduction with the DDRTree algorithm. The filtering conditions for the cell sample were cellular gene mean expression, gene dispersion ratio, mitochondrial gene expression level, and cell-cycle scores to remove these effects from the scRNA-seq data. Cell trajectory was then mapped using the “orderCells” function. For ESC subsets, we inferred the start point as the branch encompassing the previously identified EC1 subtype; for B lymphocyte subsets, the starting state was denoted as MBC; and for T lymphocyte subsets, the start point was Tn cells. The cell density of each state along the differentiation trajectory was calculated following the Monocle2 tutorial [29]. Next, DEGs over the pseudotime of cell transition were analyzed by the “DifferentialGeneTest” function in Monocle 2. In the trajectory analysis of 6 subsets of ESCs and 7 subsets of B cells, the top 50 and 100 DEGs in the re-clusters were selected, respectively. In addition, we identified specific genes with branched expression that were induced prior to lineage divergence.

### Cross-species comparison of ileum scRNA-seq data from humans, pigs, and mice

We performed a cross-species comparison integrating our dataset with the published human and mice ileum scRNA-seq datasets [66, 67]. Gene homology comparison was performed using multiple species comparison tool of Ensemble and 7029 homologous genes were replaced with identical gene names. The counting matrices were imported into Seurat for quality control. After discarding low-quality cells with ≤ 400 or ≥ 6000 expressed genes or mitochondrial genes ≥ 20%, 38,780 cells were retained. Subsequently, the data were integrated using the “IntegrateData()” functions implemented in Seurat and canonical correlation analysis (CCA) strategy based on the variable genes (variable gene = 2000, dims = 1: 30). Afterward, the data were scaled, centered, and reduced dimensionality by the aforementioned methods. Unsupervised clustering was performed and 12 cellular clusters were identified. Clusters were annotated based on canonical cell-type markers. Cell cycle and differential gene expression patterns were visualized using the same procedures as provided above. Normalized expression profiles of highly variable genes were used to compute Pearson correlation for each comparison from human-to-mice and human-to-pig was calculated separately.

### Correlative cell–cell interaction analysis

To explore potential cell–cell communication across different cell types in the PWP and NSP groups, cellular interactions analysis was performed using CellPhoneDB Python package (2.1.2) [68]. The Ensembl database was used to obtain human-homologous genes in pigs, and the ligand–receptor interactions across pig cell types were obtained through the receptor library of human-homologous genes, according to the previously described method [69]. Receptor–ligand interactions between two cell types were evaluated by expression level of the receptor in one cluster and the corresponding ligand in another cluster. Then, we obtained the most relevant interactions between ligands and receptors, and only those receptor/ligand pairs expressed at least 10% of cells within the corresponding clusters were selected.

We randomly arranged the cell clusters 1000 times to calculate the mean expression levels of receptors and ligands in the interacting clusters, which generated a null distribution for each receptor–ligand pair in the corresponding pairwise comparison of cluster–cluster crosstalk. By determining the ratio of mean greater than the actual mean, an empirical *p* value was obtained for the likelihood that the corresponding receptor–ligand complex was cell-type specific. The means of multi-subunit heteromeric complexes were calculated based on the complex member with the lowest mean expression level. We selected interactions that were biologically relevant according to the interaction pair count. Network visualization was performed using Cytoscape (Version 3.6.0), according to the obtained database of total interaction counts and connection numbers.

### Histological inflammation score and signature definition for correlated genes

To confirm the morphometric assessments of ileum inflammation, we scored samples for inflammation-related genes, using a previously published method, with some modifications [15]. Typically, we selected the following inflammation-related genes: *GZMB*, *GZMA*, *PRF*, *IFNG*, *IFNGR1*, *ISG20*, *IL4*, *IL4R*, *IL5*, *IL6*, *IL10*, *IRF2*, *IL12B*, *IL17A*, *IL17F*, *ENSSSCG00000039214*, *IL2*, *IL2RB*, *IL21*, *ENSSSCG00000023796*, *NFKBIA*, *RORA*, *RORC*, *S100A8*, *S100A9*, *STAT1*, *STAT3*, *STAT4*, *TGFB1I1*, *ENSSSCG00000038351*, and *TNF*. We scored NSP and PWP groups for these gene signatures and then calculated mean *z*-scores to combine these measurements. Significant differences were evaluated by one-sided Wilcoxon test.

We determined the mean expression level (measured as log_2_ [TPM + 1]) of 2 memory phenotype markers (*KLRB1* and *IL7R*), 5 tissue-resident markers (*RUNX3*, *CD69*, *NR4A1*, *CXCR6*, and *CD103*), 6 cytotoxicity phenotype markers (*GZMA*, *GZMB*, *PRF1*, *GNLY*, *CST7*, and *TNFSF10*), 4 exhaustion phenotype markers (*PDCD1*, *CTLA4*, *HAVCR2*, and *LAG3*), and 4 costimulatory markers (*CD28*, *CD226*, *ICOS*, and *TNFRSF9*) to determine the memory phenotype, tissue-resident, exhaustion, cellular cytotoxic, and costimulatory states of T cells.

### Gene set enrichment

DEGs across cluster subsets and designated cell types in the NSP and PWP groups were determined using the Seurat package. Log_2_ (fold change [FC]) and false-discovery rate (FDR) *q* values for DEGs were ranked after passing the control threshold: log_2_ (FC)>0.5 and *q* value<0.05. The marker genes of each cluster generally presented higher expression levels than other functional genes. We averaged gene expression over the specific clusters of each group after normalization by interrogation using the 10× Genomics Loupe Cell Browser software. Volcano plots were generated with the *X*-axis displaying log_2_ (FC) > 0.25 and the *y*-axis showing *p* value < 0.05 using GraphPad Software. Kyoto Encyclopedia of Genes and Genomes (KEGG) enrichment analysis of cluster DEGs was performed with the cluster R package clusterProfiler. The results were visualized as dot plots or bar plots using ggplot2. GO functional enrichment result was obtained using the online tools at metascape.

### Changes in cell-type compositions

Testing for differences in the proportions of cell compositions between groups (NSP vs. PWP) was conducted, as reported previously [13]. Briefly, the percentage of detection was modeled using the all cell number profiled in a corresponding piglet as an offset variable, with the condition of each sample (NSP and PWP) regarded as a covariate. The model was fitted using a General Linear Model from the R-command stats package. The associated significant differences were determined with Wald test on the regression coefficient.

### Scanning and transmission electron microscopy evaluation

We obtained electron microscopy images as described previously [70]. Ileum specimens were first fixed with 2.5% glutaraldehyde for 6 h, rinsed three times with the PBS buffer, then permeabilized with 1% osmium tetroxide for an hour, followed by briefly washing three times in PBS. The specimens were performed dehydration processing in an ethanol gradient eluting series (25%, 45%, 65%, 85%, 95%, and 100% v/v) for 15 min at each step. The specimens were incubated in absolute iso-amyl acetate for an hour, followed by dehydration in a Hitachi Model HCP-2 critical point dryer with liquid carbon dioxide. The resulting specimens were coated with gold-palladium in an Eiko Model IB5 ion coater and observed using a Hitachi TM-1000 scanning electron microscopy (Hitachi, Japan).

For transmission electron microscopy (TEM), fixation and dehydration of the ileum specimens were performed, as described. Then, the specimens were placed into a mixture of acetone and Spurr resin (v/v =1:3) for 3 h before incubating in pure Spurr resin for 8 h. Specimens were transferred into capsules with embedding medium and heated for 8 h at 65 °C. The specimens were stained with alkaline lead citrate and uranyl acetate for 10 min each and imaged using a Model H-7650 microscope.

### IF and IHC

Freshly dissected ileum sections were fixed with 4% paraformaldehyde for 6 h before embedding in paraffin and cut into 4-μm-thick sections. IF staining was performed using a modified protocol [44], as following: the specimens were deparaffinized, rehydrated, and endogenous peroxidase activity was terminated by 3% hydrogen peroxide solution. The specimens were subjected to antigen retrieval by EDTA compound (1 mol/L, pH 6.0 or 9.0) and permeabilized with 2.5% BSA for an hour at 25 °C. Next, the sections were incubated with 2.5% BSA containing the primary detection antibodies for 8 h at 4 °C, washed, and incubated for 2 h with a dilution of fluorescence-labeled secondary antibody (in PBS). Nuclei were counterstained using 120 ng/ml 4′, 6-diamidino-2-phenylindole (Cell Signaling, #8961) for 20 min before mounting in a fluorescent microscope. For IHC, the sections were incubated in AR9 buffer (pH 6.0, PerkinElmer) for antigen retrieval and heated for 20 min at 96 °C. Peroxidase was blocked before incubation with primary antibodies, and diaminobenzidine (DAB) was used for visualization. Images were acquired using a Bx63-Olympus microscope (Olympus, USA) or an LSM 510 confocal microscope for multicolor fluorescence immunostaining. Image analysis and statistic was performed using ImageJ software and the image analysis platform Fiji.

The primary antibodies were involved in IF staining: rabbit anti-*MUC2* (Biorbyt Ltd., orb372331, 1:200); rabbit polyclonal cholecystokinin (*CCK*) antibody (Origene, #20078, 1:150); mouse anti-*GCG* monoclonal antibody (Novus Biologicals, NBP2-21803, 1:2000); rabbit anti*-CD45* (intracellular domain) (Cell Signaling, #13917, 1:300); mouse anti-Sybdecan-1 (Abcam, #ab181789, 1:250); rabbit anti-IgA alpha chain (Abcam, #ab97000, 1:400); rabbit anti-*CD3* (Abcam, #ab135372, 1:200); rabbit anti-*TNF* alpha (Abcam, #EPR19147, 1:1,000); rabbit anti*-IL1β* mAb (Cell Signaling, #D3U3E, 1:500); rabbit anti-*BID* (Abcam, #ab32060, 1:100); rabbit anti-*CD3*-(E4T1B) XP® mAb (CST, #78588, 1:400); mouse anti-CD8α (RPA-T8) mAb (CST, #55397, 1:400); rabbit anti-granzyme B (D2H2F) mAb (CST, #17215, 1:800); rabbit anti-perforin (E9F7N) mAb (Cell Signaling, #44865, 1:100); and rabbit anti-*ITGAE/CD103* (EP206) mAb (Cell Signaling, #95835, 1:150). The secondary antibodies were as follows: goat anti-rabbit *IgG* H&L (Alexa Fluor® 488, Abcam, #ab150077, 1:600), goat anti-rabbit *IgG* H&L (Alexa Fluor® 555, Abcam, #ab150078, 1:400), and goat Anti-mouse *IgG* H&L (Alexa Fluor® 647, Abcam, # ab150115, 1:500).

### Enzyme-linked immunosorbent assay (ELISA)

The ileum tissue (150 mg) was added to 1.5 ml ice-cold PBS and pulverized using a tissue grinder (Tissuelyser-24, JingXin, Shanghai, China). The supernatant was extracted after centrifuging at 3000×*g* for 10 min at 4 °C. Levels of *IL10*, *IL6*, *IL12*, *IL23*, and *TGF1β* in the suspension were tested by ELISA kits (R&D Systems, USA). Supernatants were added into enzyme wells that were pre-coated with antibodies, mixed with the horseradish peroxidase (HRP)-labeled recognition antigen for 40 min at 37 °C. After resuspension with PBS containing Tween 20 (PBST), tetramethylbenzidine (TMB) was added, catalyzing a blue color that turned yellow in acid conditions. The optical density was measured using a standard enzyme reader to calculate the concentration of each molecule.

### Statistical analysis

Significant differences were assessed by Student’s two-tailed unpaired *t* test, Wald test, and Wilcoxon test, where appropriate. The GraphPad Prism V8 was used to process statistical analyses (excluding scRNA-seq data, for which we used R environment for statistical analysis). A *p* value < 0.05 was considered significant. The details of statistical methods, the value of *N*, and the representation of the data are described in respective legends.

## Supplementary information

**Additional file 1: Table S1.** Differential gene expression of different cell types in first clustering.

**Additional file 2: Fig. S1.** Primary data analysis and distribution of clusters across different samples. (a) t-SNE plot shows all cells from six samples (left) with coloring by their respective sample origin. t-SNE plot shows the different cell types before manual annotation by analyzing all cells pooled together (right). (b) Histogram shows percentage of all cell types in ileum from each sample. (c) Quantification of cell cluster proportions representation between NSP and PWP groups. No significant difference is observed in structural cells (left) and selected immune cells (right) between the NSP and PWP. Error bars: sem. Wald test. (d) Elbow plot shows ranking of the contribution of each PC to the overall variation level. (e) Variable genes in each PC, no cell-cycle related genes were observed.

**Additional file 3: Table S2.** Differential gene expression of epithelial secretory cells (ESC) subtypes in sub-clustering.

**Additional file 4: Fig. S2.** Characterizations of the main ESC subtypes and prediction of lineage differentiation related gene expression pattern. (a) Ridge-plot shows canonical marker genes across epithelial secretory cell subtypes. (b) GO analysis of EEC1 cluster-based differentially expressed genes. (c) GO analysis of B/OC cluster-based differentially expressed genes. (d) IF staining assay of number changes of goblet cell (marked by *MUC2*), and enteroendocrine cell (marked by *GCG* and *CCK*) between NSP and PWP groups (n = 6); No visual difference is observed; arrow, target cell type; scale bars, 50 μm. (e) Quantification of ESC subtype cell proportions representation between NSP and PWP groups. No significant difference is observed. (f) Graph shows the relative expression pattern of the dynamically expressed genes that follow the same trend across pseudotime.

**Additional file 5: Table S3.** GO enrichment of enteroendocrine cell (EEC)-1.

**Additional file 6: Table S4.** GO enrichment of genes related with pseudotime analysis in Fig. 2f.

**Additional file 7: Table S5.** Differential gene expression of B cell subtypes in sub-clustering.

**Additional file 8: Fig. S3.** Transcriptional modules and pseudotime define B cell and T cell subtypes. (a) t-SNE projection of B subtype cells, with each cell colored based on the relative normalized expression of selected cell type marker genes. (b) GO analysis of TC cluster-based differentially expressed genes. TC, transitory cell. (c) Heatmap shows the dynamic changes in gene expression along the pseudotime. The distribution of B subtype cells during the transition (divided into 4 phases), along with the pseudo-time. (d) GO analysis of ASC2 cluster-based differentially expressed genes. (e) Heatmap showing expression signatures of top differential expression genes in each subtype.

**Additional file 9: Table S6.** GO enrichment of genes related with pseudotime analysis in Additional **file** 8: Fig. S3c.

**Additional file 10: Table S7.** Differential gene expression of T cell subtypes in sub-clustering.

**Additional file 11: Fig. S4.** Gene expression profiles of CTLs and pseudotime analysis of T cell. (a) Dot plot shows the scaled expression of signature genes for CTL1 cells. CTL, cytotoxic T lymphocytes (b) Dot plot shows the scaled expression of signature genes for CTL2 cells. (c) Heatmap shows the dynamic changes in gene expression along the pseudotime from T naive to cytotoxic T cell.

**Additional file 12: Fig. S5.** Representative IF images of ileum tissue from NSP and PWP stained for *SDC*^+^ *IGA*^+^ ASC cells.

**Additional file 13: Fig. S6.** Differentially expressed genes and functional enrichment analysis of macrophages and monocytes. (a) Volcano plots of differentially expressed genes in macrophages from the PWP group relative to the NSP group. (b) Volcano plots of differentially expressed genes in monocytes from the PWP group relative to the NSP group. (c) Functional enrichment analysis of differentially expressed genes in macrophages between the NSP and PWP groups.

**Additional file 14: Fig. S7.** Statistical analysis of TUNEL^+^ ileum epithelial cells, differential gene expression screening and functional enrichment analysis. (a) Representative images of TUNEL-labelled ileal epithelium (green) and statistical analysis of TUNEL positive cell count per 10 villi. Scale bars, 100 μm. Student’s t test; error bars: SEM; n = 6. (b) Scatterplots show differential expression genes from EC-Im (i), ProgC (ii) and TAC (iii) in the PWP group compared with the NSP group. Selected genes related to cytochrome c are highlighted which are higher in the PWP group than the NSP group. (c) Functional enrichment analysis of differentially expressed genes between the PWP group and the NSP group.

**Additional file 15: Table S8.** Cell-cell communication analysis shows count network, interaction pairs and *p* value information.

**Additional file 16: Fig. S8.** The feature of interaction between enterocyte and immune cell. (a) Representative IHC images of ileum tissue from NSP and PWP stained for *CD103*^+^ DC cells (n= 6). Dotted area indicates more DCs migrated into villus in the PWP group compared to the NSP group. (b) Bubble plots show significant ligand-receptor pairs between ECs and other cell types in NSP and PWP.

**Additional file 17: Fig. S9.** Comparison of expression level of genes related with interaction pairs between the NSP and the PWP. (a) Expression levels of top genes *CXCL10* (ligand), *DPP4* (receptor1) and *CXCR3* (receptor2) related with DC-EC and DC-CTL interaction pairs are plotted onto t-SNE map. (b) Expression levels of top genes *TNFSF9* (ligand), *ADGRG5* (receptor1) and *TNFRSF9* (receptor2) related with DC-CTL interaction pairs are plotted onto t-SNE map. (c) Expression levels of top genes *CSF1R* (ligand) and *IL34* (receptor) related with DC-EC interaction pairs are plotted onto t-SNE map. (d) Expression levels of top genes *PTPRC* (ligand) and *MRC1* (receptor) related with DC-EC interaction pairs are plotted onto t-SNE map.

**Additional file 18: Table S9.** Differential gene expression in different cell types between the NSP and PWP groups.

**Additional file 19: Fig. S10.** Cytokines driving Th17 plasticity toward pro-inflammation state in the PWP group. (a) Bar plots show gene sets with specific function are differentially expressed in dendritic cells from the NSP group relative to the PWP group. Functional annotations are labeled by colors. (b) GO analysis of Th17 cluster-based differentially expressed genes. (c) Bubble plots show significant ligand-receptor pairs between Th17 cells and other cell types in the NSP and PWP groups. (d) Violin plot shows the expression levels of TNF in T cell subtypes in the NSP (blue) and PWP (grey) group. Unpaired two-sided Wilcoxon test. ***p < 0.001. Th17 cell is main source of TNF in ileum tissue. (e) Heatmap showing the log2 (fold change = PWP/NSP) in gene expression of the cluster-based receptor genes of TNF between the NSP and PWP groups. The elevated expressions of TNF receptor genes widely distributed in various cell types of the PWP group. (f) t-SNE plots show expression levels for cytokine genes and *TGFB* family genes in specific cell types.

**Additional file 20: Fig. S11.** Pathogenic effector CTLs are associated with mitochondrial dysfunction of structural cell in the PWP group. (a) t-SNE plot of *GZMB*, *CD3E*, *PTPRC* and *PRF1* expression levels on CTL cells. (b) GO analysis of CTL cluster-based differentially expressed genes. (c) Volcano plots of caspase family genes in the PWP group (red) relative to the NSP group (blue). No significant difference is observed between two group.

**Additional file 21: Table S10.** Cell cycle phase-specific genes.

## Data Availability

The raw sequencing data and processed next generation sequencing data have been deposited in the National Center for Biotechnology Information (NCBI) Gene Expression Omnibus (GEO) repository with accession number GSE174112 [71]. Source data are provided with this article. R markdown scripts enabling the analysis for scRNA-seq data were uploaded at https://github.com/Tangwenjie34/ScRNA-Data-Process-and-Visualization [72]. Specific codes are accessible upon request from the corresponding authors. All databases are available in the main text or the Additional Files.

## Abbreviations

scRNA-seq: Single-cell RNA-sequencing
GZMB: Granzyme B
NSP: Normal suckling piglet
PWP: Post-weaned piglets
CTL: Cytotoxic T lymphocyte
Th: T helper
t-SNE: *t*-distributed stochastic neighbor embedding
EC: Enterocytes
ESC: Epithelial secretory cell
TACs: Transient-amplifying cell
ProgCs: Progenitor cells
EEC: Enteroendocrine cell
PC: Paneth cell
B/OC: *BEST4*/*OTOP2* cell
DEG: Differentially expressed gene
MBC: Memory B cells
ASC: Antigen secretory cells
GCB: Germinal-center B cells
TC: Transitory cells
Tn: T naïve
Tfh: Tfollicular helper
Treg: T regulatory
TCM: T central memory
