# Supplementary figures and images for: Ileum tissue single-cell mRNA sequencing elucidates the cellular architecture of pathophysiological changes associated with weaning in piglets
Source: BMC Biol. 2022 May 30;20:123. doi: 10.1186/s12915-022-01321-3 (PMC9153155; doi:10.1186/s12915-022-01321-3)

**a**

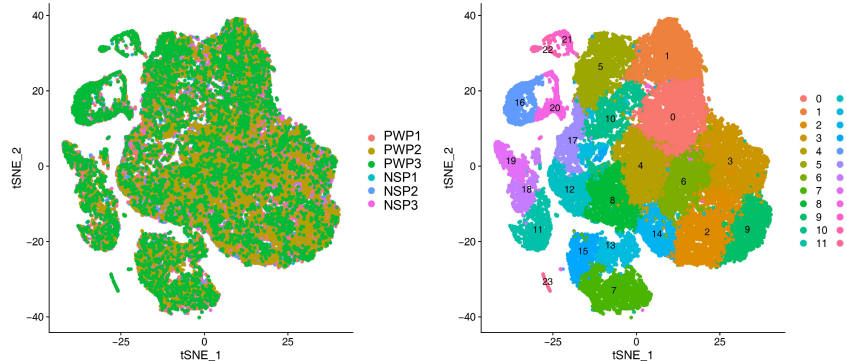

b

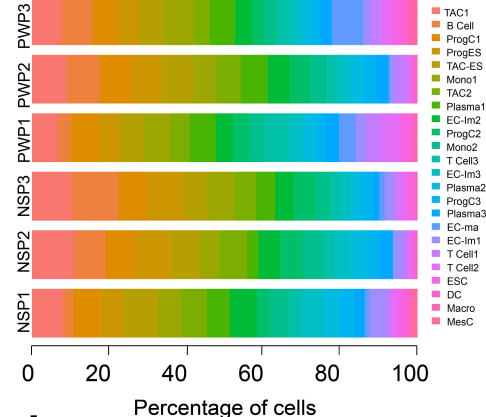

**C**

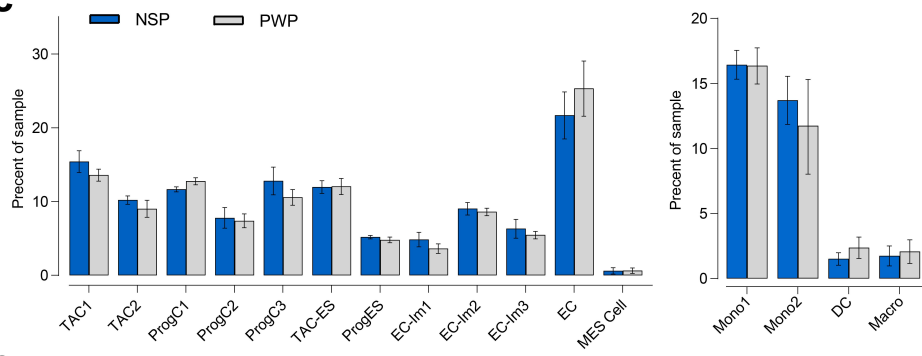

**d**

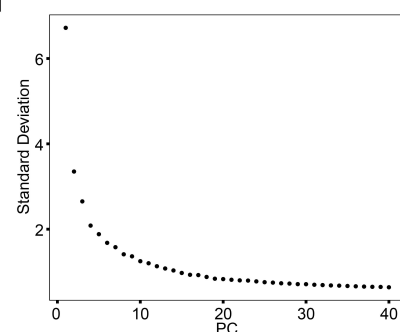

**e**

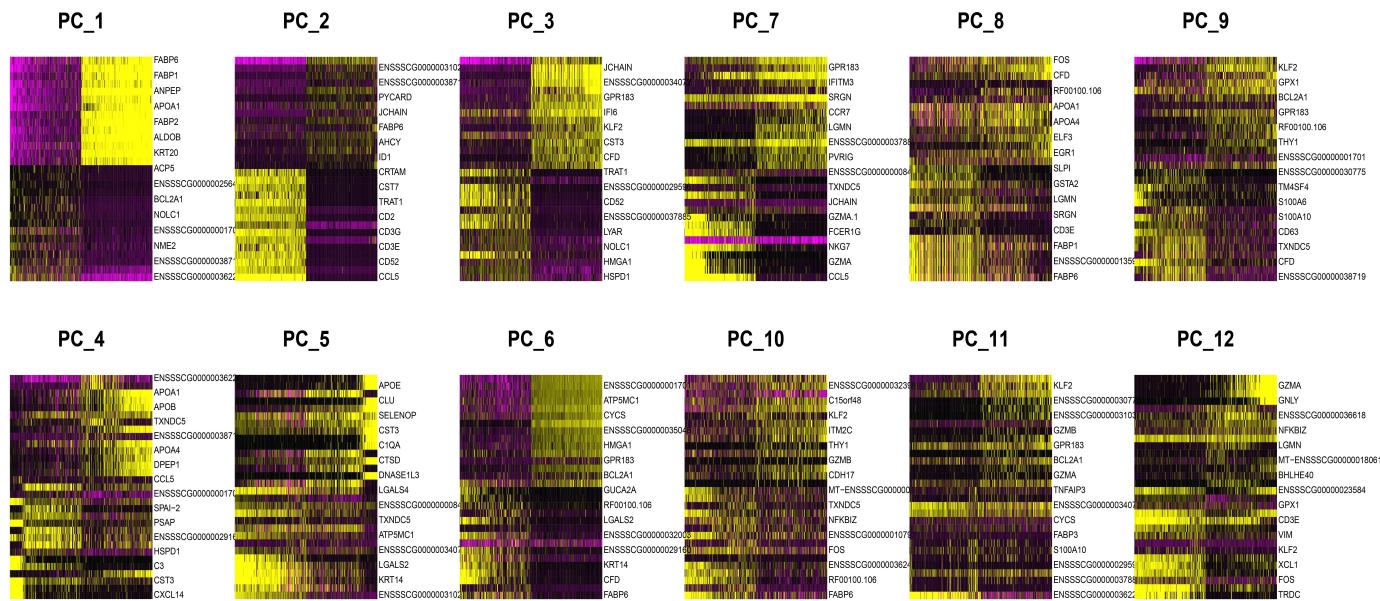

Supplement: Supplementary file 2 — Additional file 2: Fig. S1. Primary data analysis and distribution of clusters across different samples. (a) t-SNE plot shows all cells from six samples (left) with coloring by their respective sample origin. t-SNE plot shows the different cell types before manual annotation by analyzing all cells pooled together (right). (b) Histogram shows percentage of all cell types in ileum from each sample. (c) Quantification of cell cluster proportions representation between NSP and PWP groups. No significant difference is observed in structural cells (left) and selected immune cells (right) between the NSP and PWP. Error bars: sem. Wald test. (d) Elbow plot shows ranking of the contribution of each PC to the overall variation level. (e) Variable genes in each PC, no cell-cycle related genes were observed. [file 12915_2022_1321_MOESM2_ESM.pdf]

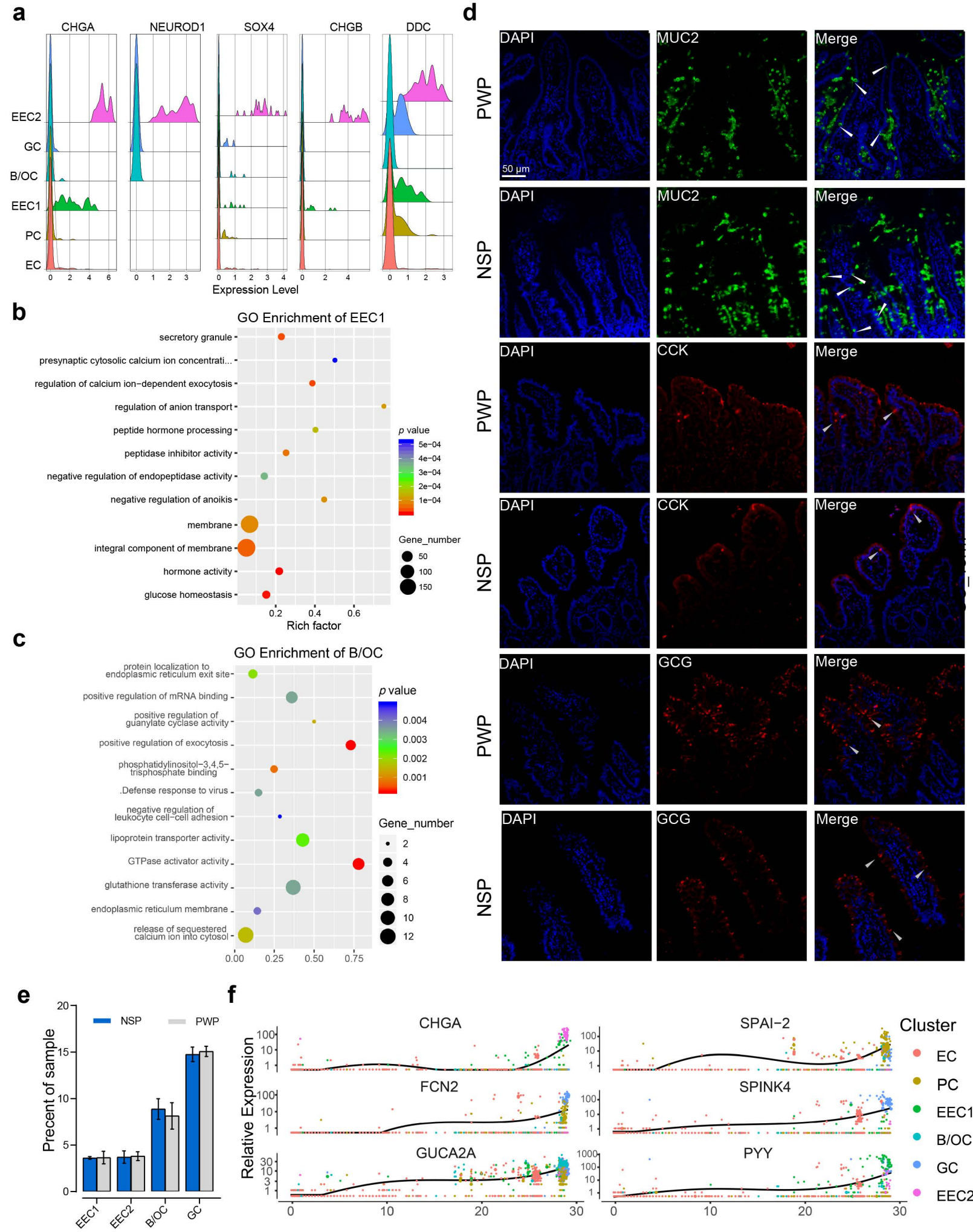

Supplement: Supplementary file 4 — Additional file 4: Fig. S2. Characterizations of the main ESC subtypes and prediction of lineage differentiation related gene expression pattern. (a) Ridge-plot shows canonical marker genes across epithelial secretory cell subtypes. (b) GO analysis of EEC1 cluster-based differentially expressed genes. (c) GO analysis of B/OC cluster-based differentially expressed genes. (d) IF staining assay of number changes of goblet cell (marked by MUC2), and enteroendocrine cell (marked by GCG and CCK) between NSP and PWP groups (n = 6); No visual difference is observed; arrow, target cell type; scale bars, 50 μm. (e) Quantification of ESC subtype cell proportions representation between NSP and PWP groups. No significant difference is observed. (f) Graph shows the relative expression pattern of the dynamically expressed genes that follow the same trend across pseudotime. [file 12915_2022_1321_MOESM4_ESM.pdf]

**a**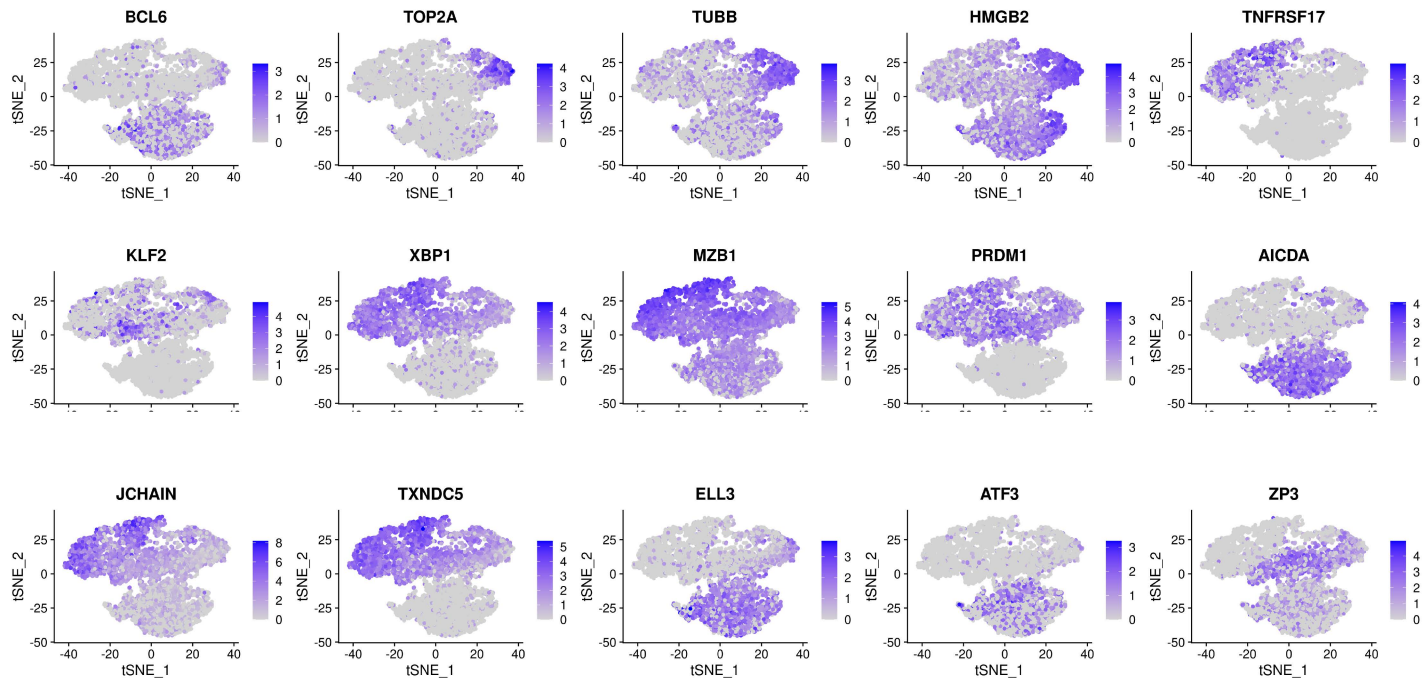**b**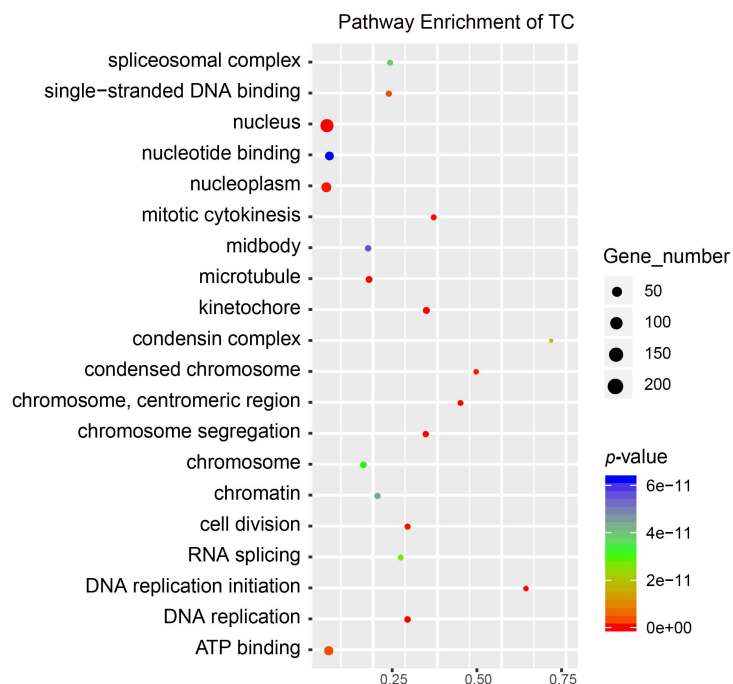**c**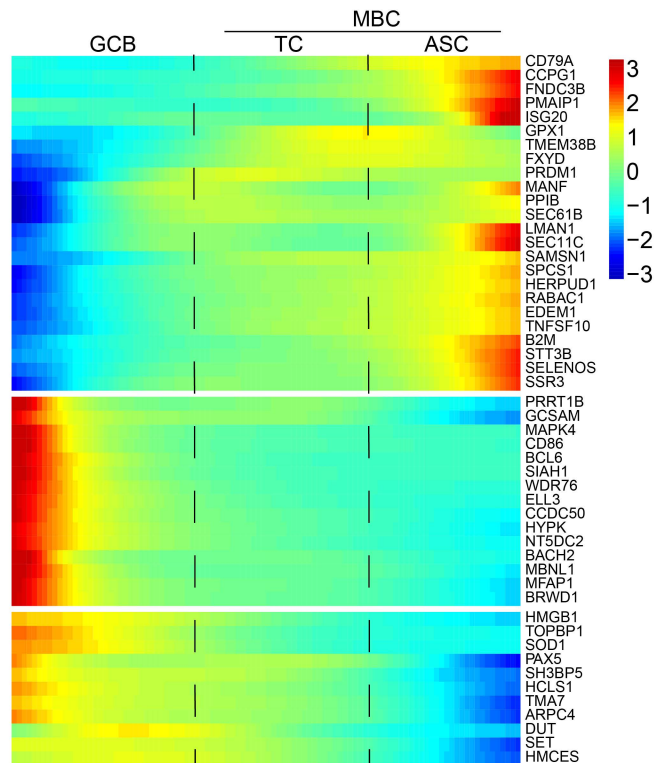**d**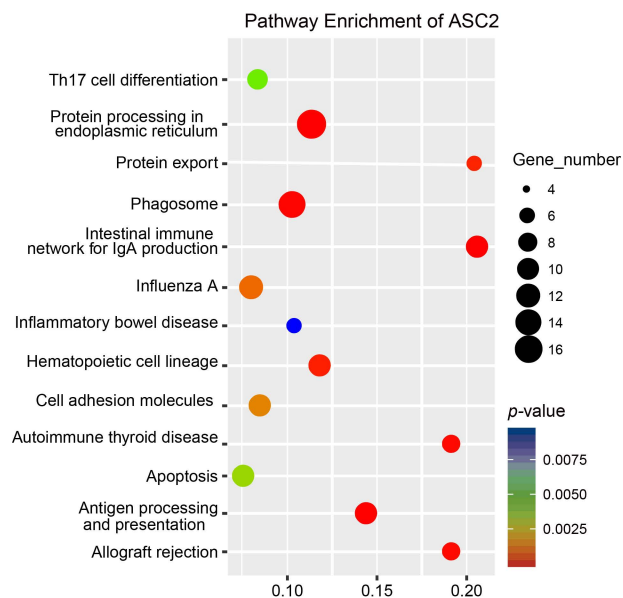**e**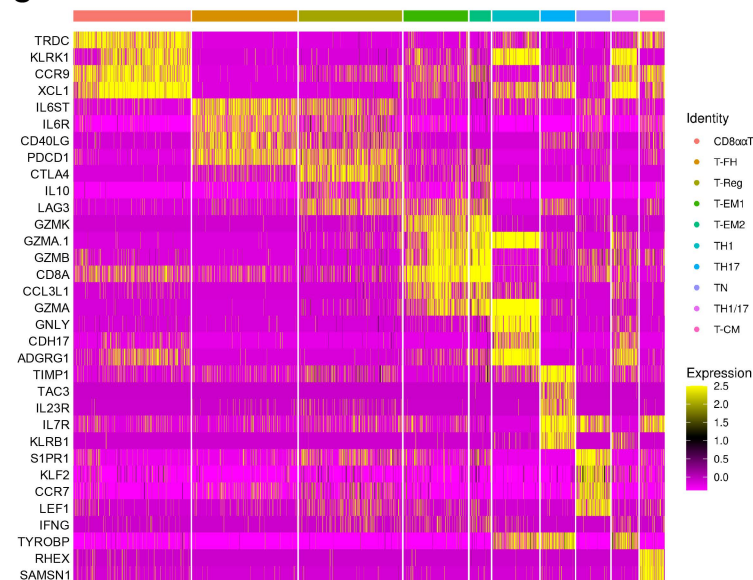

Supplement: Supplementary file 8 — Additional file 8: Fig. S3. Transcriptional modules and pseudotime define B cell and T cell subtypes. (a) t-SNE projection of B subtype cells, with each cell colored based on the relative normalized expression of selected cell type marker genes. (b) GO analysis of TC cluster-based differentially expressed genes. TC, transitory cell. (c) Heatmap shows the dynamic changes in gene expression along the pseudotime. The distribution of B subtype cells during the transition (divided into 4 phases), along with the pseudo-time. (d) GO analysis of ASC2 cluster-based differentially expressed genes. (e) Heatmap showing expression signatures of top differential expression genes in each subtype. [file 12915_2022_1321_MOESM8_ESM.pdf]

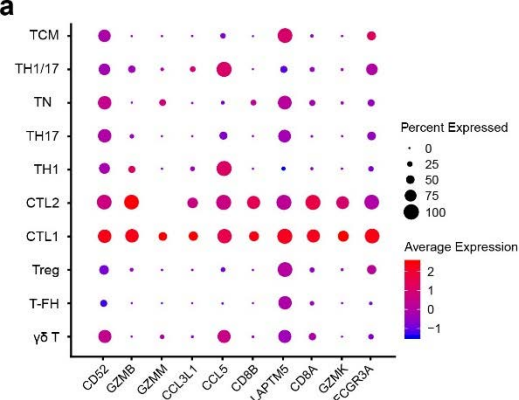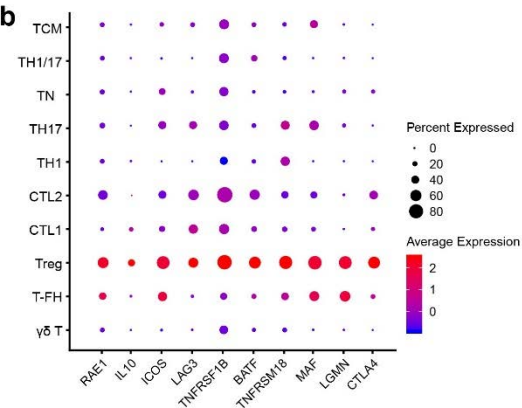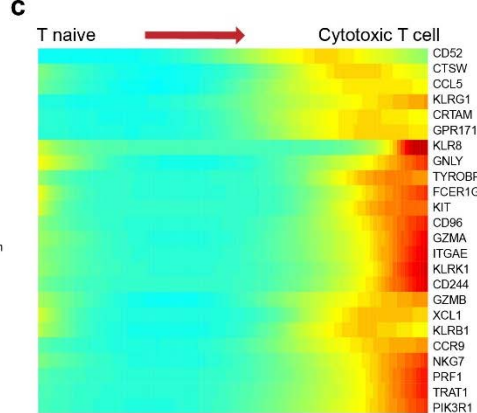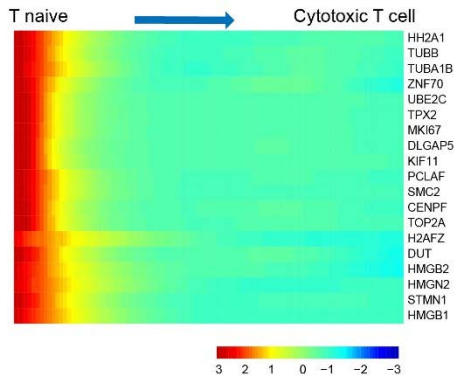

Supplement: Supplementary file 11 — Additional file 11: Fig. S4. Gene expression profiles of CTLs and pseudotime analysis of T cell. (a) Dot plot shows the scaled expression of signature genes for CTL1 cells. CTL, cytotoxic T lymphocytes (b) Dot plot shows the scaled expression of signature genes for CTL2 cells. (c) Heatmap shows the dynamic changes in gene expression along the pseudotime from T naive to cytotoxic T cell. [file 12915_2022_1321_MOESM11_ESM.pdf]

NSP

PWP

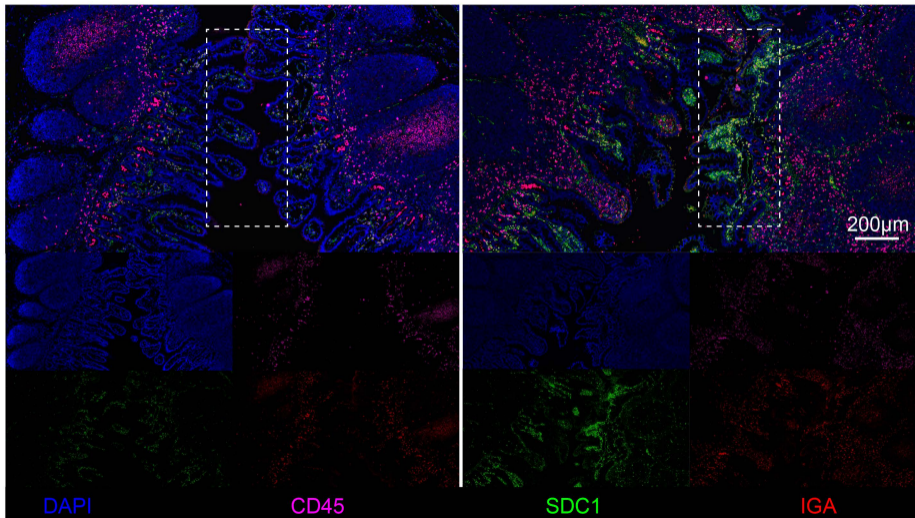

Supplement: Supplementary file 12 — Additional file 12: Fig. S5. Representative IF images of ileum tissue from NSP and PWP stained for SDC+ IGA+ ASC cells. [file 12915_2022_1321_MOESM12_ESM.pdf]

**a**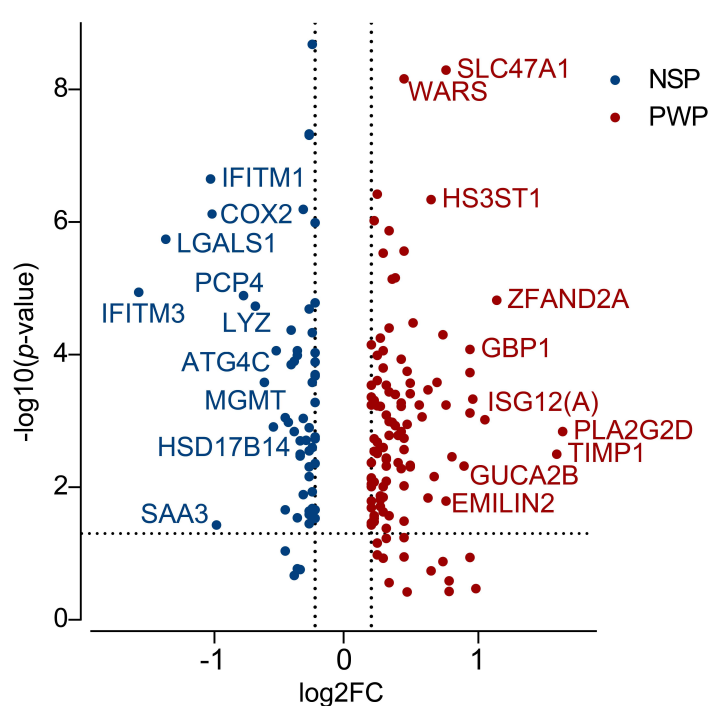**b**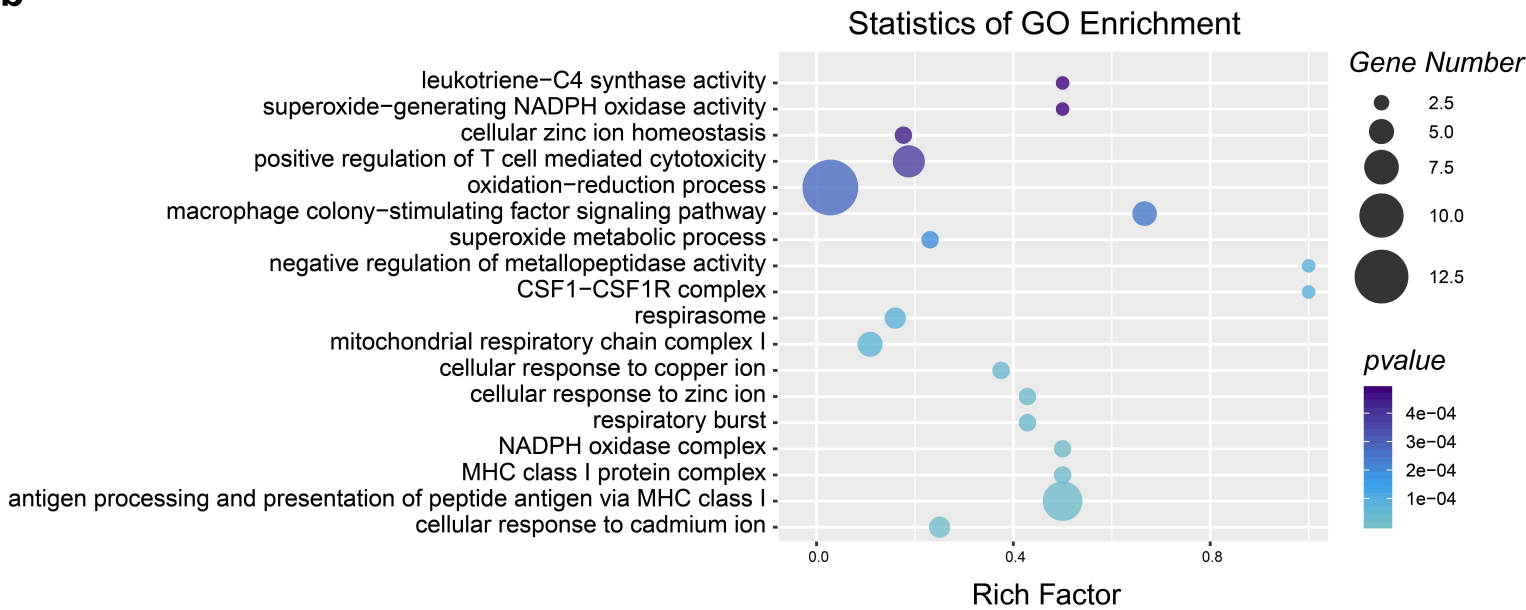**c**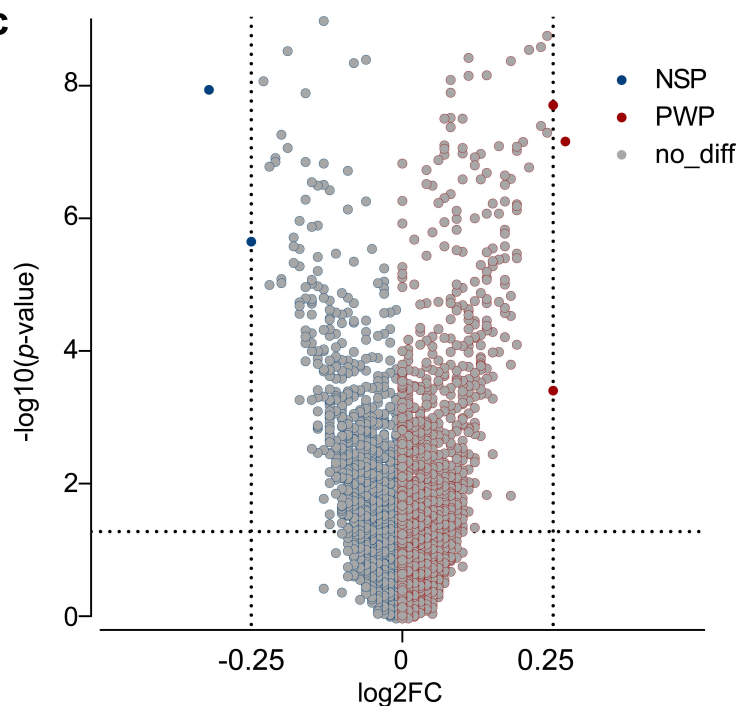

Supplement: Supplementary file 13 — Additional file 13: Fig. S6. Differentially expressed genes and functional enrichment analysis of macrophages and monocytes. (a) Volcano plots of differentially expressed genes in macrophages from the PWP group relative to the NSP group. (b) Volcano plots of differentially expressed genes in monocytes from the PWP group relative to the NSP group. (c) Functional enrichment analysis of differentially expressed genes in macrophages between the NSP and PWP groups. [file 12915_2022_1321_MOESM13_ESM.pdf]

**a**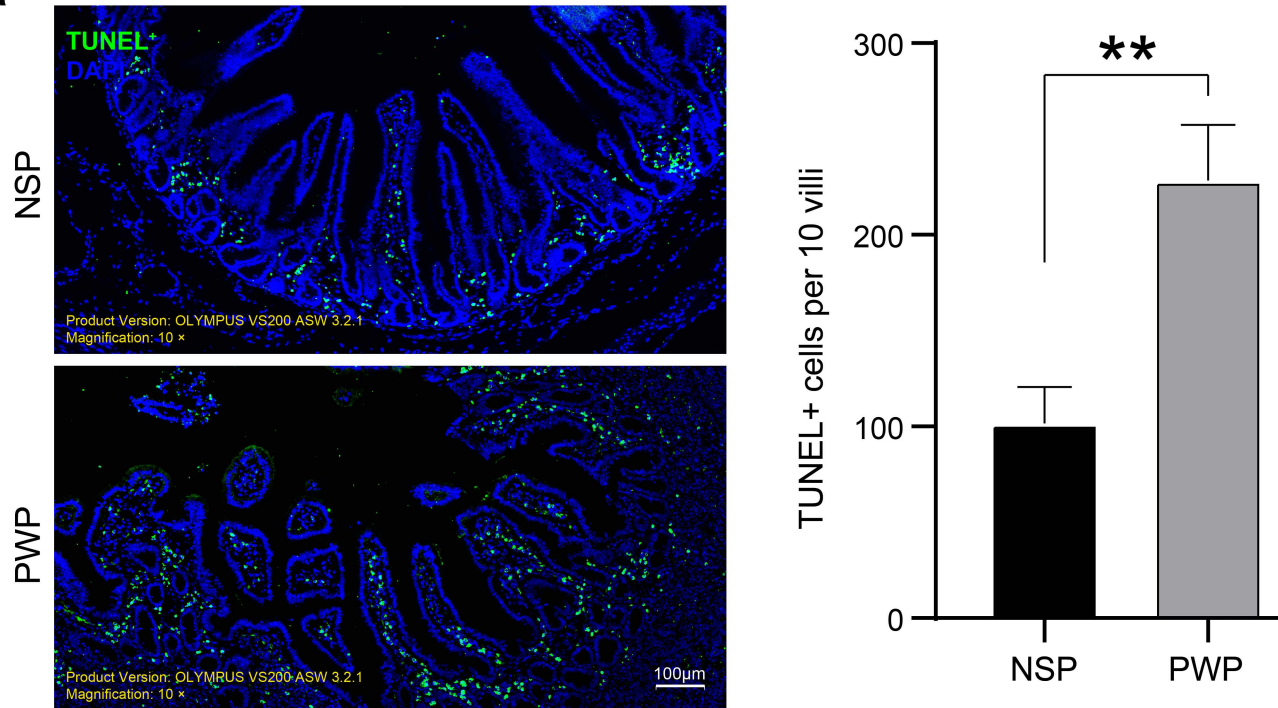**b**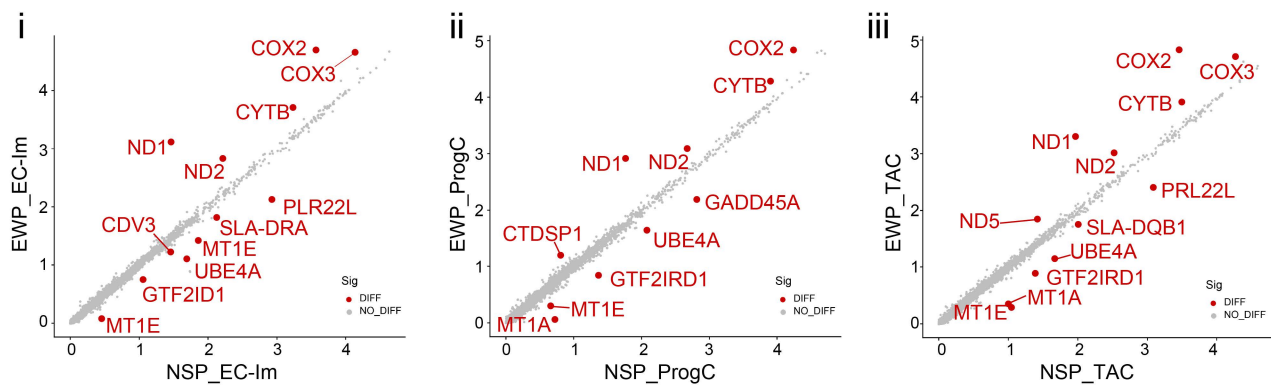**c**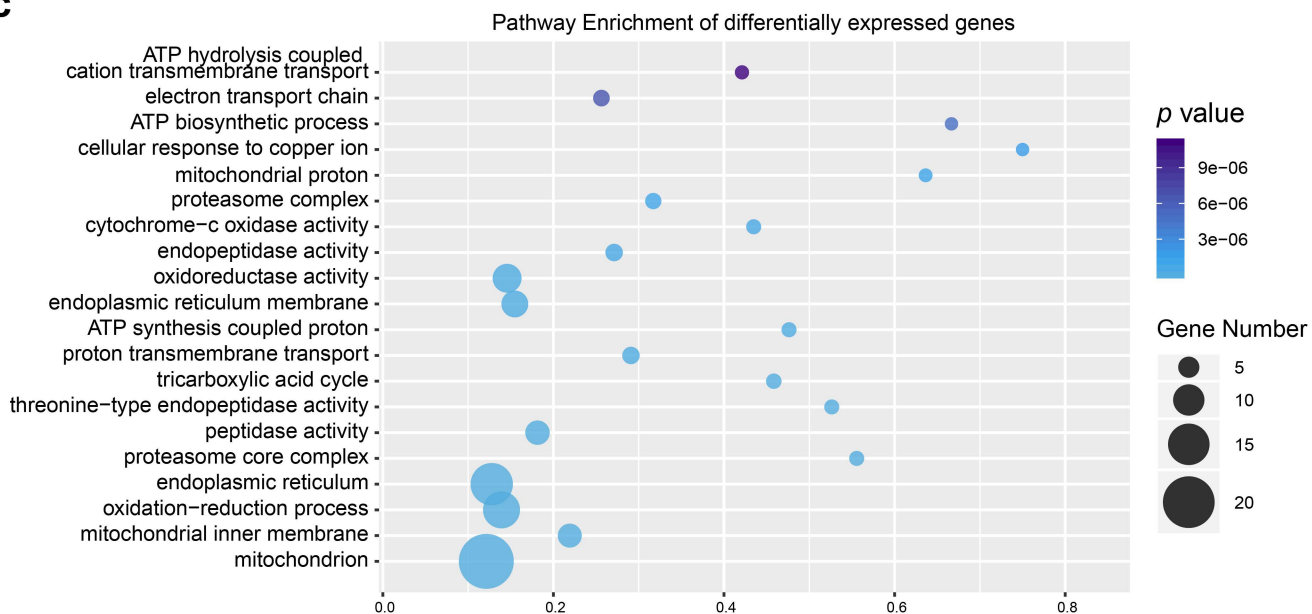

Supplement: Supplementary file 14 — Additional file 14: Fig. S7. Statistical analysis of TUNEL+ ileum epithelial cells, differential gene expression screening and functional enrichment analysis. (a) Representative images of TUNEL-labelled ileal epithelium (green) and statistical analysis of TUNEL positive cell count per 10 villi. Scale bars, 100 μm. Student’s t test; error bars: SEM; n = 6. (b) Scatterplots show differential expression genes from EC-Im (i), ProgC (ii) and TAC (iii) in the PWP group compared with the NSP group. Selected genes related to cytochrome c are highlighted which are higher in the PWP group than the NSP group. (c) Functional enrichment analysis of differentially expressed genes between the PWP group and the NSP group. [file 12915_2022_1321_MOESM14_ESM.pdf]

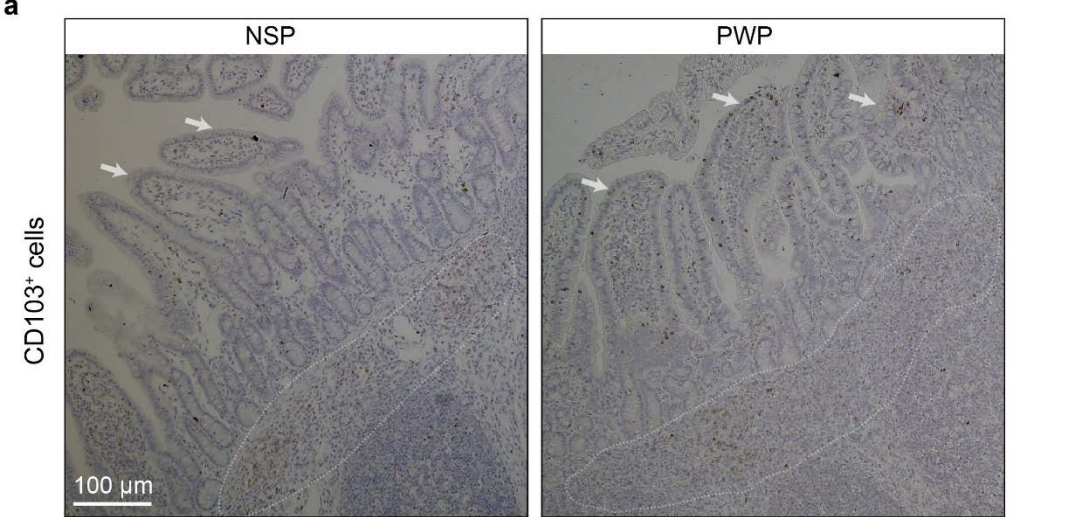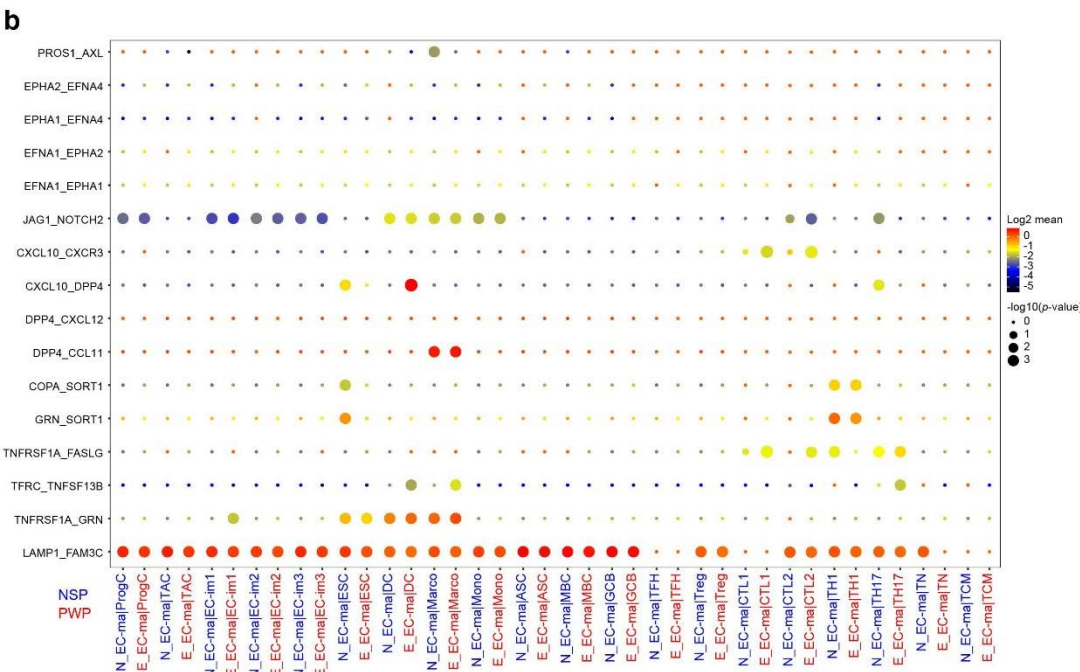

Supplement: Supplementary file 16 — Additional file 16: Fig. S8. The feature of interaction between enterocyte and immune cell. (a) Representative IHC images of ileum tissue from NSP and PWP stained for CD103+ DC cells (n= 6). Dotted area indicates more DCs migrated into villus in the PWP group compared to the NSP group. (b) Bubble plots show significant ligand-receptor pairs between ECs and other cell types in NSP and PWP. [file 12915_2022_1321_MOESM16_ESM.pdf]

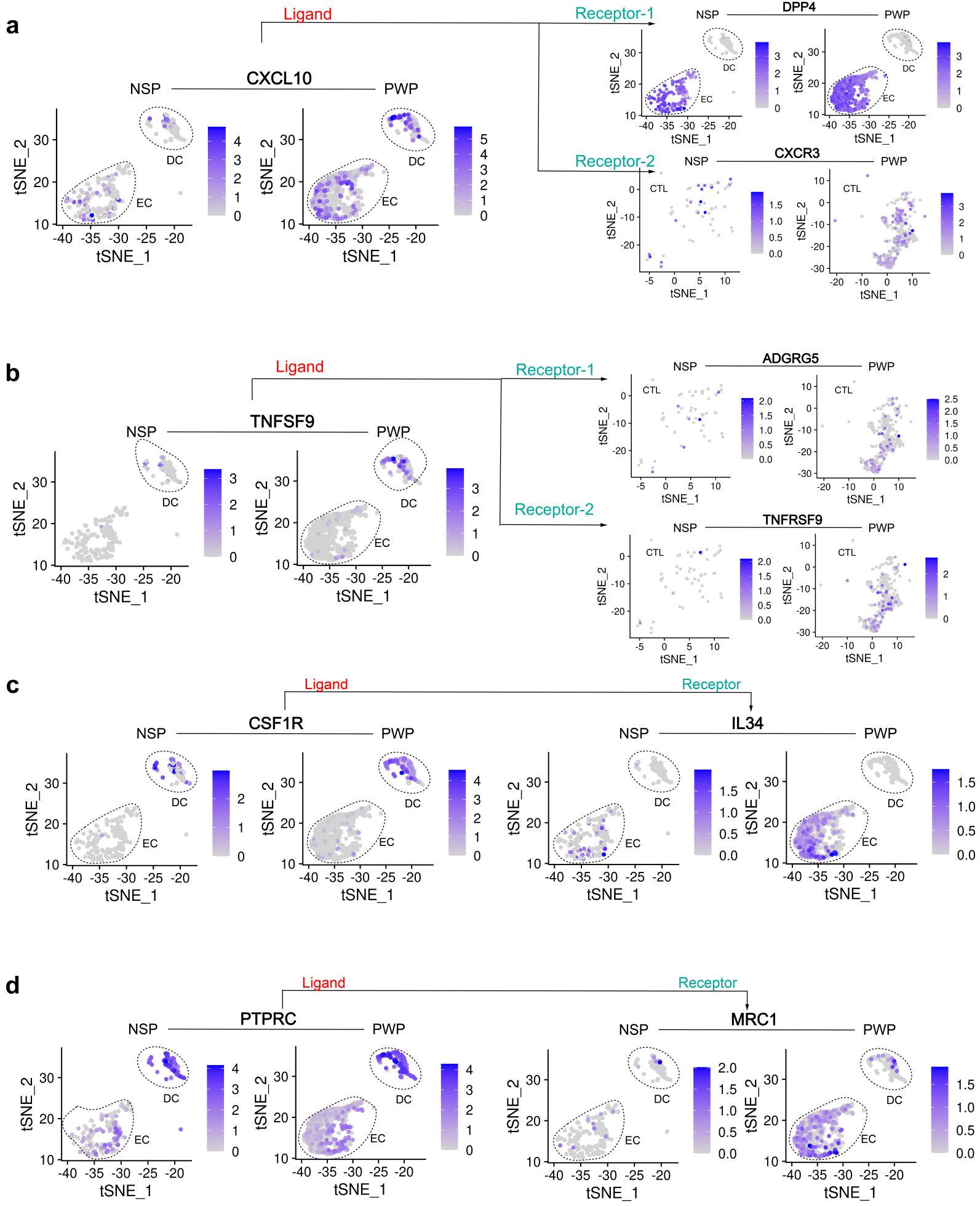

Supplement: Supplementary file 17 — Additional file 17: Fig. S9. Comparison of expression level of genes related with interaction pairs between the NSP and the PWP. (a) Expression levels of top genes CXCL10 (ligand), DPP4 (receptor1) and CXCR3 (receptor2) related with DC-EC and DC-CTL interaction pairs are plotted onto t-SNE map. (b) Expression levels of top genes TNFSF9 (ligand), ADGRG5 (receptor1) and TNFRSF9 (receptor2) related with DC-CTL interaction pairs are plotted onto t-SNE map. (c) Expression levels of top genes CSF1R (ligand) and IL34 (receptor) related with DC-EC interaction pairs are plotted onto t-SNE map. (d) Expression levels of top genes PTPRC (ligand) and MRC1 (receptor) related with DC-EC interaction pairs are plotted onto t-SNE map. [file 12915_2022_1321_MOESM17_ESM.pdf]

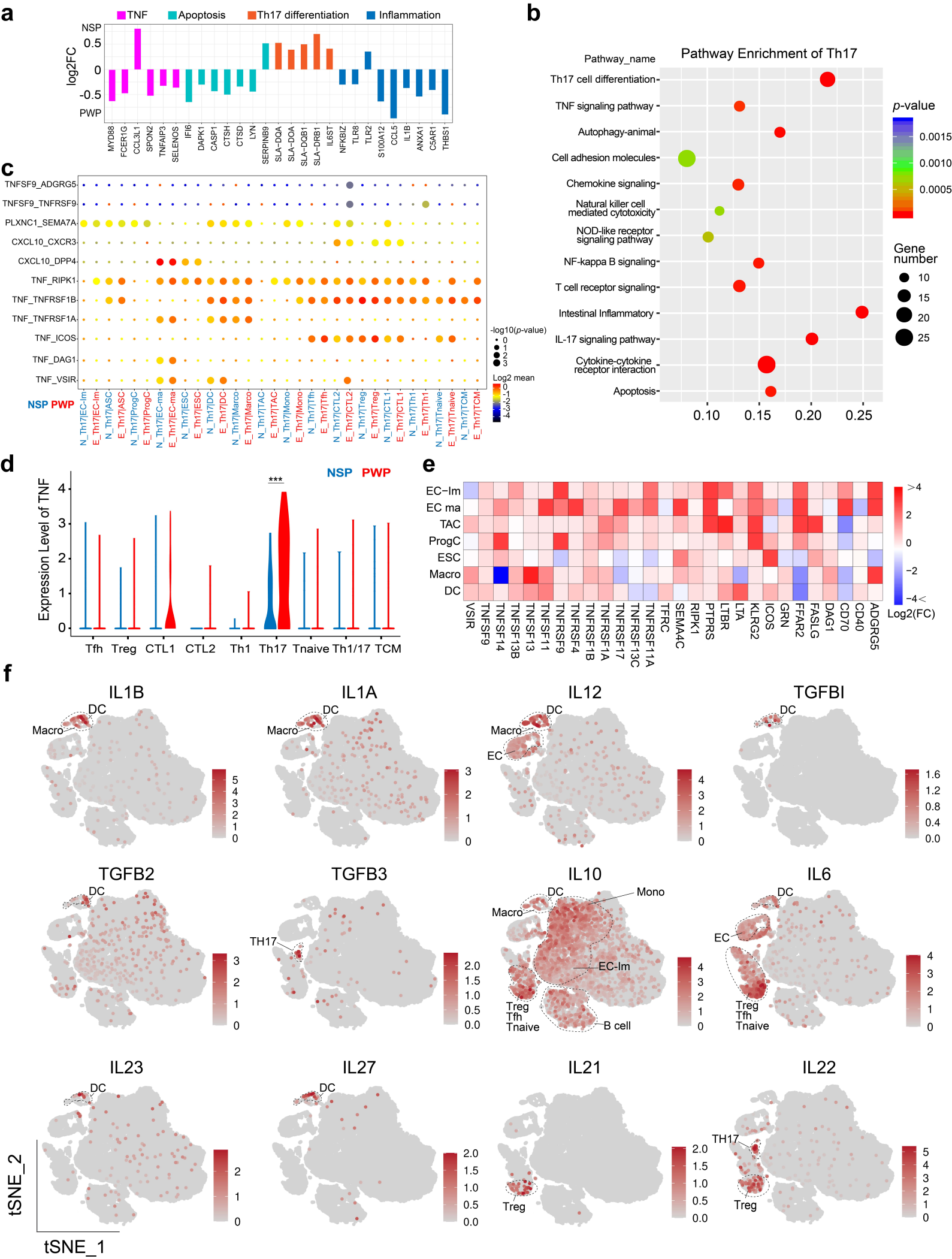

Supplement: Supplementary file 19 — Additional file 19: Fig. S10. Cytokines driving Th17 plasticity toward pro-inflammation state in the PWP group. (a) Bar plots show gene sets with specific function are differentially expressed in dendritic cells from the NSP group relative to the PWP group. Functional annotations are labeled by colors. (b) GO analysis of Th17 cluster-based differentially expressed genes. (c) Bubble plots show significant ligand-receptor pairs between Th17 cells and other cell types in the NSP and PWP groups. (d) Violin plot shows the expression levels of TNF in T cell subtypes in the NSP (blue) and PWP (grey) group. Unpaired two-sided Wilcoxon test. ***p < 0.001. Th17 cell is main source of TNF in ileum tissue. (e) Heatmap showing the log2 (fold change = PWP/NSP) in gene expression of the cluster-based receptor genes of TNF between the NSP and PWP groups. The elevated expressions of TNF receptor genes widely distributed in various cell types of the PWP group. (f) t-SNE plots show expression levels for cytokine genes and TGFB family genes in specific cell types. [file 12915_2022_1321_MOESM19_ESM.pdf]

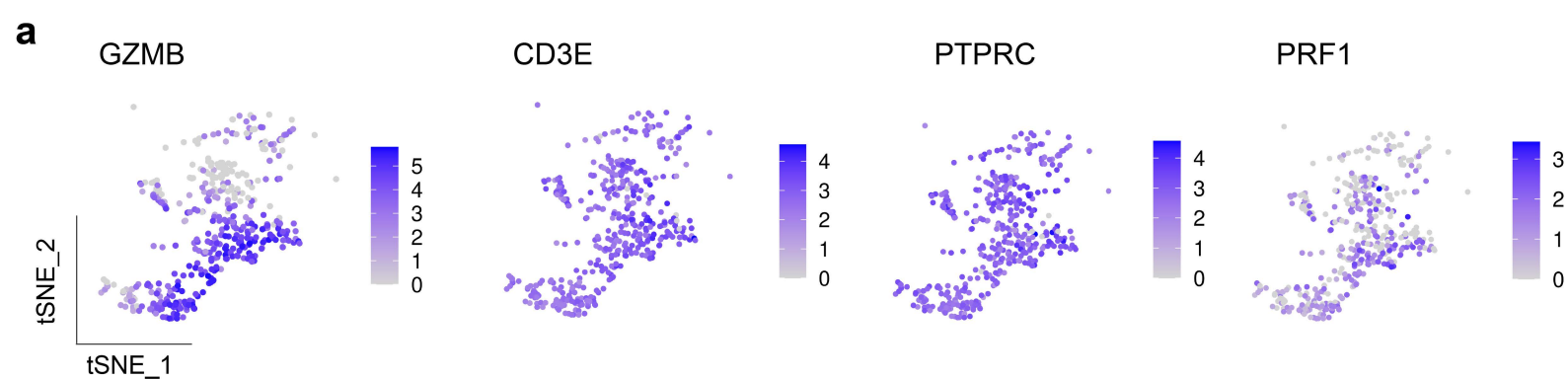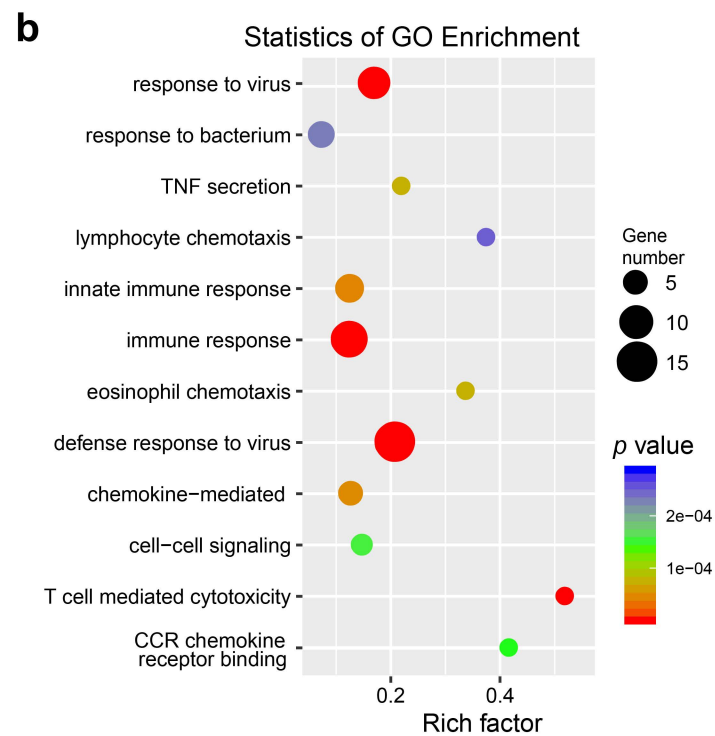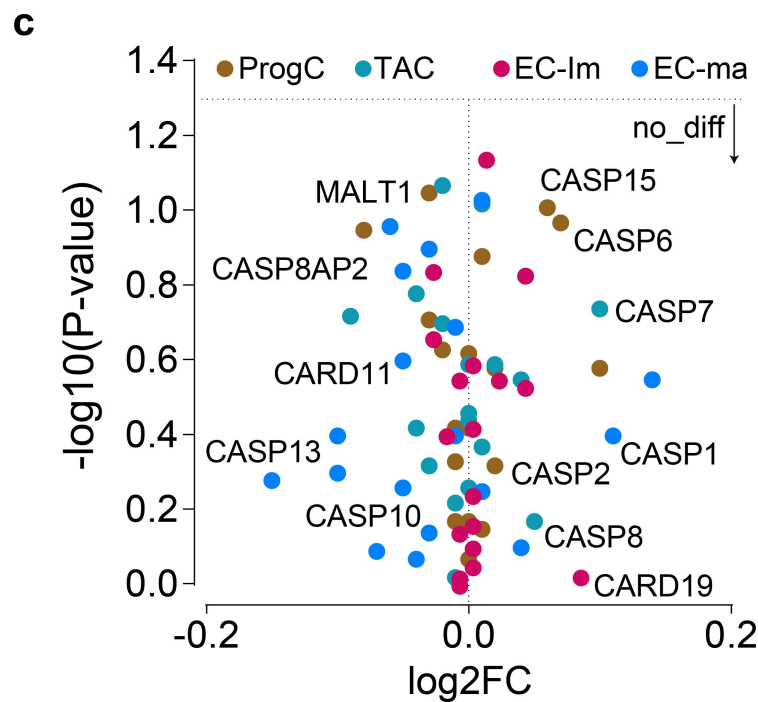

Supplement: Supplementary file 20 — Additional file 20: Fig. S11. Pathogenic effector CTLs are associated with mitochondrial dysfunction of structural cell in the PWP group. (a) t-SNE plot of GZMB, CD3E, PTPRC and PRF1 expression levels on CTL cells. (b) GO analysis of CTL cluster-based differentially expressed genes. (c) Volcano plots of caspase family genes in the PWP group (red) relative to the NSP group (blue). No significant difference is observed between two group. [file 12915_2022_1321_MOESM20_ESM.pdf]
